# Clonotypic analysis of protective influenza M2e-specific lung resident Th17 memory cells reveals extensive functional diversity

**DOI:** 10.1038/s41385-022-00497-9

**Published:** 2022-03-08

**Authors:** Ajibola Omokanye, Li Ching Ong, Cristina Lebrero-Fernandez, Valentina Bernasconi, Karin Schön, Anneli Strömberg, Mats Bemark, Xavier Saelens, Paulo Czarnewski, Nils Lycke

**Affiliations:** 1 Mucosal Immunobiology and Vaccine Center (MIVAC), Department of Microbiology and Immunology, Institute of Biomedicine, University of Gothenburg, Gothenburg, Sweden; 2 VIB-UGent Center for Medical Biotechnology, VIB, Ghent, Belgium and Department of Biomedical Molecular Biology, Ghent University, Ghent, Belgium; 3 Department of Biochemistry and Biophysics, National Bioinformatics Infrastructure Sweden, Science for Life Laboratory, Stockholm University, Solna, Sweden

## Abstract

The fate of tissue-resident memory CD4 T cells (Trm) has been incompletely investigated. Here we show that intranasal, but not parenteral, immunization with CTA1-3M2e-DD stimulated M2e-specific Th17 Trm cells, which conferred strong protection against influenza virus infection in the lung. These cells rapidly expanded upon infection and effectively restricted virus replication as determined by CD4 T cell depletion studies. Single-cell RNAseq transcriptomic and TCR VDJ-analysis of M2e-tetramer-sorted CD4 T cells on day 3 and 8 post infection revealed complete Th17-lineage dominance (no Th1 or Tregs) with extensive functional diversity and expression of gene markers signifying mature resident Trm cells (*Cd69*, *Nfkbid*, *Brd2*, *FosB*). Unexpectedly, the same TCR clonotype hosted cells with different Th17 subcluster functions (IL-17, IL-22), regulatory and cytotoxic cells, suggesting a tissue and context-dependent differentiation of reactivated Th17 Trm cells. A gene set enrichment analysis demonstrated up-regulation of regulatory genes (*Lag3, Tigit, Ctla4, Pdcd1*) in M2e-specific Trm cells on day 8, indicating a tissue damage preventing function. Thus, contrary to current thinking, lung M2e-specific Th17 Trm cells are sufficient for controlling infection and for protecting against tissue injury. These findings will have strong implications for vaccine development against respiratory virus infections and influenza virus infections, in particular.

## Introduction

Pre-existing influenza-specific CD4 T cells have been shown to correlate with reduced morbidity in humans and in murine models of influenza virus infection CD4 T cells can convey protection independently of specific antibodies.^1–6^ To achieve strong protection against influenza virus infection many studies have identified mucosal vaccination and in particular intranasal (i.n) immunization to be superior to systemic immunization for the generation of lung resident T cells.^7–11^ Indeed, lung resident memory CD4 T cells have been found to be critical for protection by functioning as helper cells for lung B - and CD8 T cell responses, but also as effector cells exerting direct actions.^7,12–15^ Whereas influenza-specific Th1 cells have been associated with resistance against infection in the past, several recent studies have documented a critical role also for Th17-lineage cells in providing antibody-independent protection.^5,16–20^ However, other studies have documented a detrimental role of Th17 cells for protection against infection, which is reminiscent of their proinflammatory functions observed in several autoimmune diseases.^21–23^ Nevertheless, the exact mechanisms for protection still awaits to be determined, but, for example, lung infiltration of neutrophils was dependent on IL-17 and IL-17 also plays an essential role in regulating transport of secretory IgA (SIgA) across the mucosal membrane.^5,24,25^ Furthermore, Th17 cells have been reported to attract influenza-specific cytotoxic CD8 T cells to the site of infection and they appear critical for the development of iBALT.^18,22,26–30^

Direct evidence that influenza-specific CD4 T cells can play a critical role for immune protection against infection was obtained by using an adoptive transfer model with HA-specific Th1 or Th17 memory CD4^+^ T cells.^6,31^ Interestingly, whereas the transferred CD4^+^ T cells supported CD8^+^ T cell and antibody responses, they also acted as cytotoxic CD4 T cells, in a perforin-dependent manner.^9,31^ Indeed, it was the tissue-resident memory CD4 T cells (Trm) that exhibited cytotoxicity against influenza virus-infected cells.^31–34^ These CD4 Trm cells were thought to be derived from Th1 cells that undergo maturation to T helper CTL (ThCTL) functions, while memory Th17 cells have not been associated with a cytotoxic function.^35,36^ However, the critical importance of Th1 cells for protection against influenza virus infection was recently challenged when it was found that Tbet-deficient mice exhibited unperturbed protection against infection in the complete absence of Th1 cells and a sizeable fraction (20%) of *Tbx21*-defective CD4 T cells produced IL-17 in the lung.^20^ Interestingly, an IL10-deficient mouse model revealed that influenza viruses appear to have evolved mechanisms to inhibit Th17 responses, thus, potentially representing a mechanism of immune evasion.^18,37–39^ In addition, Th17 cells have been shown to play a key role in preventing opportunistic secondary bacterial lung infections, an often lethal complication that is frequently associated with influenza virus infections.^16,30^

Plasticity among CD4 T cell subsets has attracted much attention in recent years.^40–43^ For example, the ability of Th1 cells to transform into Th17 or Treg cells has been amply documented. Co-expression of Tbet or RORγt by Foxp3-regulated cells; IL10 production by Th1, Th2, and Th17 cells; IFNγ production by Th17 cells; and IL13 production by Th1 cells, are other examples of functional plasticity.^44–46^ However, the mechanisms that drive this plasticity in antigen-committed CD4 T cells are still largely unknown, although they appear to be heavily context-dependent and may be ascribed to tissue-specific factors.^47^ In fact, little is known about the cues that promote Trm cell development following immunizations, let alone immunizations for long-term influenza-specific protection.^48^ Swain and co-workers identified a critical checkpoint at days 5-7 post-infection when effector CD4 T cells developed into Trm cells, which required IL-2 and cognate antigen-presentation.^49,50^ A critical question to answer is what the nature is of the Trm cell if it develops from effector cells and how can the Trm cell provide a progeny with necessary effector functions to combat infection and protect against tissue injury? For example, are Trm cells derived from Th1 effector cells, required for protection against influenza virus infection, or can these be derived from Th17 cells, especially in the light of unperturbed resistance against infection in *Tbx21-* deficient mice?^20,51–53^ Indeed, Th17-skewed memory CD4 T cells have been reported to exhibit a mix of Th1, Th2, Th17, and Treg-functions, even including cytotoxic features, in response to viral infection and inflammation.^44,45^ Thus, despite a growing appreciation of lung Trm cells for immune protection against influenza virus infection the signaling and transcriptional pathways that regulate the function of these cells during infection remain elusive.

A vaccine targeting conserved viral proteins may be the key to developing cross-reactive, or “universal” protection against influenza.^6^ The ectodomain of matrix protein 2 (M2e) is a 24 amino acid peptide sequence that is highly conserved across influenza A virus subtypes.^54,55^ A wealth of studies document the capacity of M2e-based vaccines to confer protection in mouse models of infection, with phase I clinical studies confirming both safety and immunogenicity in humans.^54^ We have developed an intranasal vaccine candidate against influenza A virus infections and have provided strong evidence in support of its broadly protective ability in the mouse model.^5^ The CTA1-3M2e-DD fusion protein conferred protection via induction of both anti-M2e antibody and lung-resident M2e tetramer-specific CD4 T cells, which were predominantly Th17 cells.^5^ Significant protection was also observed in antibody-deficient mice following i.n immunizations, while loss of protection was found in IL-17-deficient mice, despite the presence of comparable anti-M2e IgG antibody titers as in wild-type (WT) Balb/c mice.^5^ Moreover, in the absence of M2e-specific CD4 T cells congenic H-2^b^ restricted BALB.B mice were poorly protected, again while anti-M2e serum antibody titers were similar to those in well-protected Balb/c mice.^5^ In the present study we sought to capture the functions of lung M2e-specific Th17 Trm cells at the gene transcriptional level during the course of infection. We performed a temporal transcriptome and TCR VDJ-analysis of M2e-specific Trm cells isolated from the lung by FACS-sorting on day 3 and 8 post infection, in mice that had been immunized i.n 2 and 6 months earlier with CTA1-3M2e-DD.

## Results

### Lung resident CD4 T cells are critically important for protection against influenza virus infection in M2e-immunized mice

**Fig. 1:**
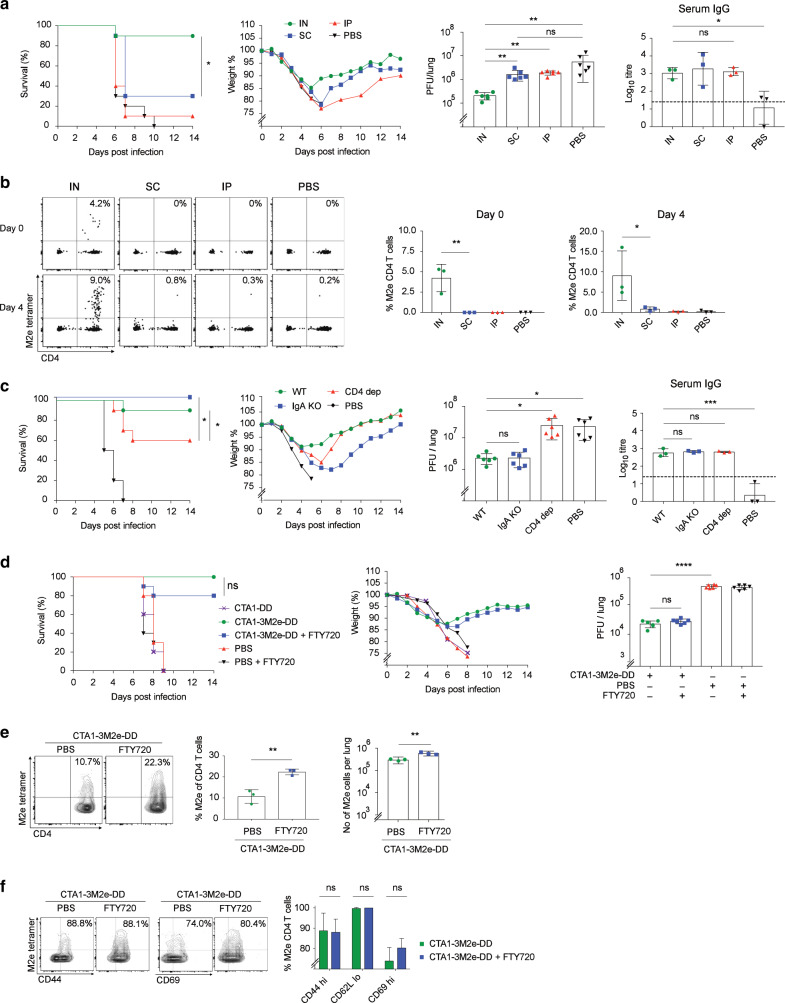
Intranasal immunizations with CTA1-3M2e-DD stimulate resident memory M2e-specific CD4 T cells and protection against infection in the lung. Female Balb/c mice, 6–8 weeks old, were immunized i.n., s.c., or i.p. with 5 µg of CTA1-3M2e-DD on days 0, 10, and 20 or given PBS alone i.n.at the indicated time points. After 3 weeks the mice were infected i.n with 4xLD_50_ of the influenza X47 virus strain and monitored for survival and loss of body weight for 14 days. **a** Survival and body weight in groups of mice (*n* = 10), with lung virus titres measured in duplicate at 4 days post infection (*n* = 3). Mice were euthanized if the weight loss ≥ 30%. IgG anti-M2e serum antibody titers were detected by ELISA and given as log_10_ titers ± SD in the different groups on day 0 of challenge. **b** Flow cytometric analysis of lung CD4 T cells for binding to the M2e-tetramer prior to and 4 days post infection (*n* = 3). Representative FACS plots (gated on 7AAD^−^, CD19^−^, F4/80^−^, CD8α^−^ lymphocytes) show M2e-specific CD4^+^ T cells at indicated time points **c** Wild-type, IgA^−/−^ (KO) or CD4 T cell-depleted WT mice, all on a Balb/c background, were immunized i.n and challenged by X47 virus as in A. Effective CD4 T cell depletion was achieved by i.p-injections of 400 µg anti-CD4 mAb (clone GK1.5) administered on 3 consecutive days prior to infection. Survival and body weight were monitored (*n* = 10), with lung virus titres measured in duplicate at 4 days post infection (*n* = 3). IgG anti-M2e serum antibody titers were detected by ELISA and given as log_10_ titers± SD in the different groups on day 0 of challenge. Results are given as mean values ± SD for each group. **d** One day prior to infection, 100 µg FTY720 or PBS was administered i.p. and then every 48 h during the experiment. Mice were euthanised if the weight loss ≥ 30%. Survival and body weights were monitored in the different groups (*n* = 10), with lung virus titres measured in duplicate 4 days post infection (*n* = 3). **e** Representative FACS plots (gated on 7AAD^−^, CD19^−^, F4/80^−^, CD8α^−^ lymphocytes) show M2e-specific CD4^+^ T cells on day 14 in the lung of immunized mice with (blue) or w/o (green) FTY720 blockade (*n* = 3). **f** Lung M2e-specific CD4^+^ T cells in WT (green) or FTY720 treated (blue) mice on day 14 from i.n immunized mice labeled with anti−CD44, −CD62L and −CD69 Mabs and analyzed by FACS. The Log-rank test was applied for survival comparisons, one-way ANOVA test with Tukey’s correction for virus titers, log_10_ antibody titers, and comparisons of M2e-specific CD4 T cell frequencies by FACS. Significance was denoted by *p ≤ 0.05, **p ≤ 0.01, ***p ≤ 0.001, or ****p ≤ 0.0001 and ns; not significant. These data show one representative experiment out of 3 independent experiments giving similar results.

We have previously shown that M2e-specific lung Trm cells were effectively generated through i.n immunizations with the CTA1-3M2e-DD fusion protein and strong protection against a lethal challenge infection with a heterosubtypic influenza virus strain was observed.^5,11,56,57^ Here we asked if the route of immunization influenced the level of protection and to what extent M2e-specific CD4 T cells were directly involved. We used Balb/c mice that were challenged 3 weeks after i.n immunizations with a 4xLD_50_-dose of the X47 virus strain (H3N2). Only i.n immunizations with CTA1-3M2e-DD resulted in significant survival rates and reduced morbidity, while subcutaneous (s.c) or intraperitoneal (i.p) immunizations failed to protect the animals, albeit comparable serum anti-M2e IgG antibody titers were detected in all groups (Fig. 1a). The better protection corresponded to a significant reduction in lung virus titres after i.n immunizations, which also was the only route to give M2e-tetramer specific CD4 T cells in the lungs (Fig. 1a, b). Noteworthy, the infection itself failed to induce detectable M2e-specific CD4 T cells in unimmunized control mice (Fig. 1b). Hence, the presence of M2e-specific lung CD4 T cells was a consequence of successful local i.n immunization, which was much superior to systemic immunizations (s.c, i.p or i.m), as we have also documented before.^11^

While serum IgG anti-M2e antibody levels were shared by all immunized mice we investigated whether lack of local IgA antibodies could have accounted for the better protection found in i.n immunized mice. To this end we asked whether IgA^-/-^ deficient mice were equally or less well protected compared to WT mice.^58^ We found that both strains were equally well protected following i.n immunizations and serum anti-M2e IgG titres were comparable in WT and IgA^-/-^ mice (Fig. 1c). By contrast, when CD4 T cells were depleted from WT mice by administration of anti-CD4 mAb prior to the challenge infection we observed significant reduction in survival in immunized mice (Fig. 1c). This correlated with a reduced ability to control virus propagation (Fig. 1c). The M2e-peptide did not stimulate CD8 T cell responses and so depletion of CD8 T cells had no effect on protection or virus load in i.n immunized and challenged mice (Supplementary Fig. S1a-c).^5,11,56,59^ Taken together, lung CD4 T cells, but not mucosal IgA, was required for reducing virus replication, which was also associated with enhanced immune protection.

**Fig. 2:**
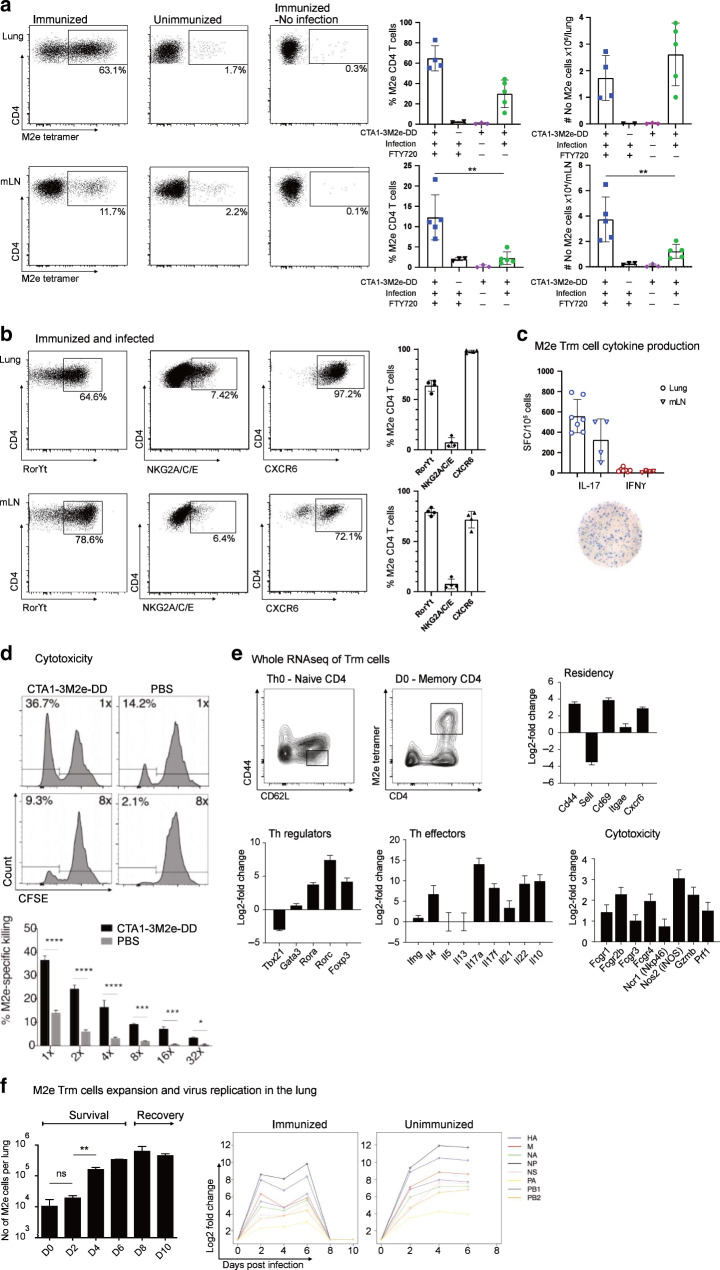
M2e-specific Th17 Trm cells in the lung demonstrate cytotoxicity and IL-17 production. Female Balb/c mice, 6-8 weeks old, were immunized i.n with 5 µg of CTA1-3M2e-DD or CTA1-DD on days 0, 10, and 20 or given PBS alone i.n. to generate lung resident M2e-specific Trm cells. The phenotype and function were determined in M2e-tetramer binding CD4 T cells isolated from the mediastinal lymph node (mLN) or the lung at 2 (**a**–**c**) or 6 months (**d**–**f**) after the priming immunization. Mice were treated with FTY720 after inoculation of live virus infection as described in Fig.1d, unless stated otherwise. The mice were infected i.n with a sublethal dose (**a**–**c**) or 4xLD_50_ (**f**) of the influenza X47 virus strain, as indicated. (**a**, **b**) Flow cytometry analysis of the distribution and absolute number of M2e-tetramer binding CD4 T cells among all CD4 T cells at 8 days in lungs and mLN following infection, or no infection, in mice i.n immunized 2 months earlier or unimmunized control mice. Assessments in (**a**) were also done in the absence of FTY720 blockade, as indicated. **d** In vitro cytotoxicity was determined in freshly isolated lung CD4 Trm cells from mice rested for 6 months following i.n immunizations and taken 4 days after infection with 4xLD50 influenza X47 virus. CD4 T cells were extracted by negative selection from pooled lung tissue and co-cultured at serial dilutions with a 1:1 mix of peptide-pulsed CFSE^lo^ (0.4 μM) and non-pulsed CFSE^hi^ (1.0 μM) spleen-derived B cells for 6 h at 37 °C before analysis by FACS. **e** M2e-tetramer binding CD4 T cells were isolated from the lungs at 6 months after i.n immunizations and whole RNA-seq analysis was performed in pooled tissues (*n* = 2 × 3). The relative expression levels given as Log2-fold changes ± SD of specified sets of selected genes in M2e-specific Trm cells were compared to the levels of expression of these genes in naive splenic CD4 T cells (CD62L^high^, CD44^−^, CD69^−^) is shown. Differential expression of selected genes associated with different functions; tissue residency, Th-lineage committment, and effector cytokine production are given as mean values ± SD. **f** Increased numbers of lung M2e-tetramer^+^ Trm cells following infection as determined by FACS on indicated days in mice i.n immunized 6 months prior to the experiment (left panel). Lungs were harvested and total mRNA extracted from whole tissues (*n* = 2) at indicated days following infection. An RNA-seq analysis was performed of differential expression of influenza X47 virus transcripts as indicated in lung tissue from unimmunized or i.n immunized mice at 6 months following i.n immunizations. Unimmuized mice were sacrificed or died due to infection before day 8. Mean values ± SD are given for each group and Log-rank test was applied for survival comparisons, one-way ANOVA test with Tukey’s correction for virus titre comparison, and unpaired, two-tailed *t*-test for FACS population comparisons. *p ≤ 0.05, **p ≤ 0.01, ***p ≤ 0.001, ****p ≤ 0.0001 or ns; not significant. These data show one representative experiment out of 2 (**a**–**c**, **e**, **f**) or 3 (d) independent experiments giving similar results.

Next we addressed whether lung resident CD4 T cells were sufficient for protection or if recruitment of CD4 T cells from the lymph nodes or spleen to the lung was required.^15^ FTY720-treatment was used to block egress of CD4 T cells from lymph nodes.^60^ To confirm that M2e-tetramer binding cells were lung resident CD4 T cells we injected labeled anti-CD45.1 Mabs i.v and found isolated cells to be negative, as also reported earlier (Supplementary Fig. S1d).^5,61^ Indeed, the FTY720-treatment had little effect on protection against a lethal challenge infection with X47 virus (Fig. 1d). Lung virus titres were essentially unchanged despite FTY720-treatment (Fig. 1d). Of note, protection was critically dependent on the M2e-peptide as no protection was observed in mice immunized with the CTA1-DD adjuvant vector alone (Fig. 2d).^5^ The distribution of M2e-specific T cells among all CD4 T cells in the lung post-infection was two-fold higher in FTY720-treated (22%) as compared to untreated i.n immunized mice (11%) (Fig. 1e). The great majority of M2e-specific CD4 T cells were CD44^+^ (90%) and CD69^+^ (80%) cells irrespective of FTY720-treatment (Fig. 1f). Thus, it appeared that M2e-specific lung Trm cells do not require support from lymph node or spleen M2e-specific effector memory CD4 T cells to convey strong protection.^10^

### Long-term protection correlates with the presence of Th17 Trm cells in the lung

To assess M2e-specific resident CD4 T cell memory (Trm) cells mice were rested for 2 or 6 months following the last day of i.n immunizations. We then challenged these mice by a sublethal dose of X47 virus to decipher which functional memory CD4 T cell subsets that expanded in the lung and in the mediastinal lymph nodes (mLN) in response to infection. FTY720 was used to block recruitment of memory CD4 T cells from lymph nodes and the spleen to the lung as before.^53^ The frequency of M2e^+^ Trm cells was significantly higher in the lung compared to memory CD4 T cells in mLN (Fig. 2a). Whereas FTY720-blockade did not affect the level of M2e^+^ CD4 T cells in the lung, it strongly increased M2e^+^ CD4 T cells in the mLN, as expected (Fig. 2a). Uninfected immunized mice exhibited very low levels of M2e-binding CD4 T cells in the lung and mLN after 2 months, attesting to the great potential of local expansion of these cells following infection (Fig. 2a). The vast majority of M2e^+^ memory CD4 T cells in lung and mLN were Th17 cells (Rorγt^+^) (65–80%) as determined by flow cytomtry. A reproducible fraction of the M2e^+^ CD4 T cells expressed the NKG2A-marker, associated with a cytotoxic function (7% NKG2A^+^) and more lung cells were CXCR6^+^ (>95%) than in mLN (70%), a marker previously found to correlate with Trm-mediated anti-infectious protection in the lung (Fig. 2b).^62^ When tested in antigen-recall responses in vitro the M2e^+^ memory CD4 T cells from lung and mLN exhibited a clear dominance of IL-17 spot forming cells (SFC), with low to undetectable levels of IFNγ SFCs found (Fig. 2c) Clear evidence of specific cytotoxicity was also observed in lung M2e^+^ Trm cells when incubated with M2e-labeled target B cells in vitro (Fig. 2d). Thus, following a challenge-infection in i.n CTA1-3M2e-DD immunized mice we found a dominant memory M2e-specific Th17 (Rorγt^+^) response in the lung and mLN that was associated with IL-17 and cytotoxic functions.

A detailed characterization of gene expression in lung M2e-specific Trm cells at 6 months after i.n immunizations, prior to a challenge-infection, was performed. Following isolation of lung M2e^+^ Trm cells from pooled mice the results from whole RNA-Seq analysis was compared to a similar analysis obtained with naïve splenic CD4 T cells (CD62L^hi^CD44^lo^ CD4^+^) (Fig. 2e). Fold-changes in gene expression were compared and as expected, the lung M2e-specific Trm cells exhibited higher levels of memory and tissue-residency genes, *CD44*, *CD69*, and *Itgae* (CD103), as well as increased chemokine receptor *CXCR6* gene expression (Fig. 2e).^62^ A Th-lineage gene analysis revealed a strong presence of Th17-defining genes (*Rorc* and *Rorα*), while Th1 or Th2 master gene regulators (*Tbx21* and *GATA3*) were not expressed (Fig. 2e).^30^ Moreover, lung M2e-specific Trm cells expressed distinctly higher levels of *IL17a*, *IL17f*, *IL21* and *IL22* cytokine genes, but also *IL-4* and *IL-10* gene expression were up-regulated, compared to that observed in naive CD4 T cells (Fig. 2e).^63,64^ There was also a higher expression of the *Foxp3* gene, which could indicate the presence of regulatory T cells (Tregs), but which could also be consistent with Th17 cells.^65^ Also, in agreement with the NKG2A-flow cytometry finding, genes associated with cytotoxicity were up-regulated in M2e^+^ Trms (Fig. 2b, e).

Following a challenge-infection with X47 virus we monitored the expansion of M2e^+^ Trm cells in the lung of FTY720-treated mice to get an estimate of the proliferative capacity of these cells in response to infection. We observed a 100-fold increase in lung M2e^+^ Trm cells over the 8–10 days of infection (Fig. 2f). This dramatic increase correlated closely with a reduction in virus propagation, as assessed by fold-change of influenza virus segment-specific transcripts in the lungs of immunized as compared to unimmunized control mice (Fig. 2f). This suggested a critical window for protection between days 2 and 4 of the infection in i.n immunized mice.

### Single-cell analysis reveals several subsets of M2e-specific Th17 Trm cells during infection

**Fig. 3:**
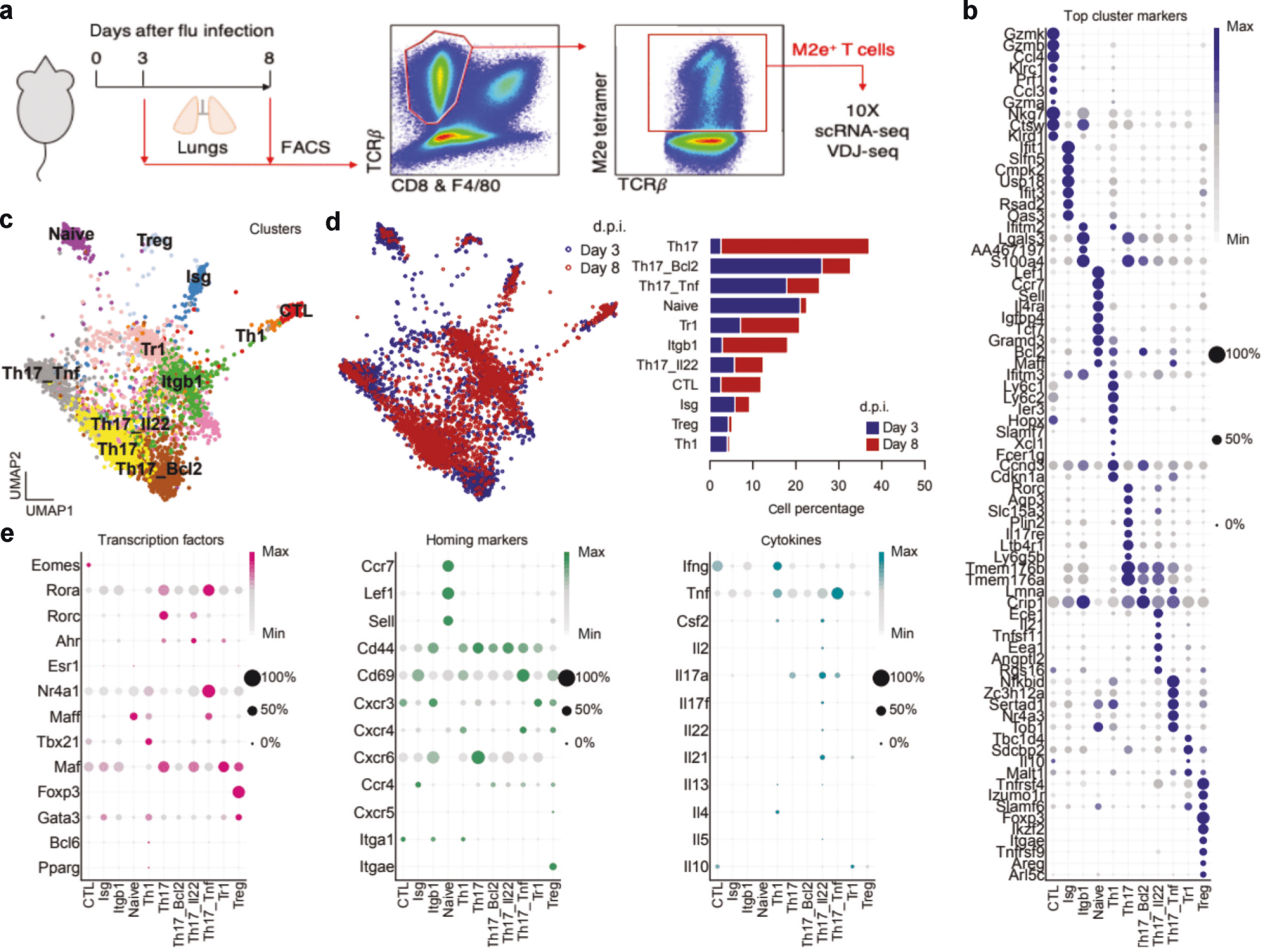
Single cell RNAseq transcriptomic analysis of M2e-tetramer binding Trm cells following infection. A schematic representation of the protocol and FACS gating strategy used for scRNAseq analysis of lung M2e-specific Trm cells from mice i.n immunized 6 months earlier with CTA1-3M2e-DD. **a** Samples were isolated from 5 pooled mice on day 3 or 8 post-inoculation with 4xLD_50_ of the influenza X47 virus strain. Cells were sorted by FACS and gated on M2e-tetramer^+^ and TCRβ^+^ cells and processed for 10X Chromium single cell library preparation. **b** Defining the cluster identity by dot plot representation of the expression levels of the top marker genes found in each cluster. Darker shades of blue indicate higher expression and circle sizes represent the percentage of cells expressing a certain gene in the clusters. **c** The UMAP embedding showing Leiden clustering of M2e-binding CD4^+^ T cells sampled from days 3 and 8 and identified by Th-lineage defining genes. **d** The break-down of the UMAP representation in **b** into day 3 or 8 post-inoculation samples (left panel). Distribution in % of M2e-binding CD4^+^ T cells in the major clusters and separated on days 3 (blue) or 8 (red) post-inoculation (right panel). **e** Dot plot expression levels of genes encoding transcription factors, homing markers, and cytokines linked to CD4^+^ T cell functions are given for each cluster, as in **b**.

To get a detailed understanding of the expansion and functional differentiation of the responding lung CD4 Trm cells following infection we performed single-cell RNA-Seq (scRNAseq) analysis of TCRβ^+^ M2e-tetramer-binding FACS-sorted cells on days 3 and 8 post-infection, correlating with protection and decreased viral load (day 3 effector phase), and increased survival (day 8 recovery phase), respectively. At each time point we sorted the M2e^+^ Trm cells to high purity (>95%) from 5 pooled mice that had been immunized 6 months earlier and analyzed their transcriptomes at the single-cell level using the 10X Chromium platform (Fig. 3a). After quality control, a total of 2013 cells from day 3 and 3262 cells from day 8 were used for downstream analysis (Supplementary Fig. S2a–c). First, we confirmed that all sequenced CD4 T cells expressed *Cd3e*, *Cd3g*, *Thy1*, and *Cd4* genes, and that expression of the *Cd8a* and *Cd8b* genes was absent from our samples (Supplementary Fig. S2a). In total, 11 clusters were identified using Louvain algorithm (Fig. 3b, c, and Supplementary Fig. S2b). Differentially expressed genes (DEG) across clusters allowed us to identify major T cell subsets and based on *Cd44, Sell*, and *Ccr7* gene expression we could distinguish a single cluster with naïve cells and 10 clusters hosting different memory CD4 T cell subsets (Fig. 3b–e, Supplementary Fig. S3, and Data S1). The most striking observation was that 4 clusters could be directly assigned to the Th17-lineage based on their higher expression of the *Il17a*, *Rorc,Rorα, Tmem176a/b*, and *Ahr* genes (Fig. 3b, c, e, Supplementary Fig. S4i–n).^66^ Of these an archetypical Th17 cluster (Th17) exhibited the highest expression of *Serpinb1a*, *Znrf1*, *Il17re*, and *Ramp1*, suggestive of a homeostatic expansion of these cells (Supplementary Fig. S3b, d).^67^ A second Th17 cluster (Th17/IL-22) expressed tissue repair genes *Il22*, *Il17f*, and *Csf2* (GM-CSF) and was also high in *IL-21* gene expression (Fig. 3b, e). Further, a third cluster (Th17/Tnf) expressed high levels of activation/inflammation genes such as *Tnf*, *Nr4a1*, and *Nfkbid* and hosting increased expression of *Malt1*, *Maff*, and *Cxcr4* genes, similar to Th1 cells and possibly associated with a pro-inflammatory function (Fig. 3b, e, Supplementary Fig. S3a, d).^68^ Finally, we identified a Th17 cluster (Th17/Bcl2) that exhibited elevated expression of S-phase genes, *Bcl2* and *Lmna*, and that was lower in expression of cytokine and activation genes, possibly potential memory cells (Fig. 3b, e). Less prominent clusters hosted cells with gene expression signatures typical for Tregs; *Foxp3*, *Il2ra* (CD25) and *Itgae* (CD103), Type 1 regulatory T cells (Tr1); *Il10*, *Lag3* and *Tigit* and Th1 cells; *Ifng*, *Tbx21* (Tbet) and *Icam1* (Fig. 3b, e, Supplementary Fig. S4c–e). Importantly, we found a single cluster hosting cytotoxic CD4 T cells (ThCTL) typified by *Eomes*, *Gzma*, and *Ccl4* gene expression, and which also expressed several anti-viral encoding genes; *Klrc1*, *Klrd1*, *Prf1*, and *Nkg7* (Fig. 3b, e, Supplementary Fig. S4f).

One cell cluster (Itgb1) expressed *Itgb1*, *Ctsd*, and *Iftim3* genes and shared expression of many genes observed in other clusters, albeit at lower levels, but these cells also had higher proliferative S and G2M gene expression, in line with an intermediate state of differentiation (Fig. 3b, e, Supplementary Fig. S4h). Finally, we identified a cluster that expressed the *Ifih1*, *Cmpk2*, and *Daxx* genes and which was also highly enriched for interferon-stimulated genes (Isg) (*Ifit3, Rsad2,Gbp4, and Isg15*), identified as the Isg cluster (Fig. 3b, e, Supplementary Figs. S3d, S4g). Both the Itgb1 and Isg clusters shared high expression of *Tmem176a/b* genes, linking these clusters to Th17-lineage cells, and suggesting that we, in fact, had identified a total of 6 Th17-lineage related clusters (Fig. 3b, Supplementary Fig. S3d).^69^ Therefore, we concluded that at least 70–75% (day 3 plus 8 samples) of the M2e-binding CD4 T cells in the UMAP embedding appeared to be the progeny of activated lung Th17 Trm cells.

Strikingly, samples taken from day 3 and 8 were represented in all clusters, albeit differentially distributed (Fig. 3d). However, noteworthy, the naïve, Th1, and Treg clusters were predominantly found in day 3 samples and were probably contaminating cells (Fig. 3d). Th17/Bcl2 (memory) and Th17/Tnf (pro-inflammatory) clusters, associated with early antiviral response, were observed in day 3 samples (Fig. 3d). By contrast, the Th17-, Th17/IL-22- as well as the Tr1-cluster were more strongly represented in day 8 samples, corresponding to the recovery phase of infection with more need for anti-inflammation and tissue repair (Fig. 3d). Indeed, the computed top DEG, typifying each cluster on day 3 in the effector phase of infection, hosted several interferon-induced anti-viral genes (*Irf1*, *Irf7*, *Ifrd1*, *Socs1*, *Socs3, Ifit1, Bst2*, and *Isg15*) as well as genes associated with mature resident Trm cells (*Cd69*, *Nfkbid*, *Brd2*, *FosB*) (Fig. 3b,e, Supplementary Fig. S5a and Data S2). From the early response of M2e-specific Trm cells to the recovery phase of infection on day 8 there was a shared increase in expression of adaptive anti-viral response genes (*Nkg7*, *Ccl5*, *Ctsd*, *Ctsw*) and lymphocyte activation and maturation-associated genes (*Icos*, *Jak3*, *Id2*, *Ramp1*, *Maf*, *Lgals1, Lgals3*) as well as genes linked to cell motility (*Cxcr6*, *Ccr2*, *Itgb2*, *Lsp1*, *Glrx*)(Fig. 3e, Supplementary Fig. S5b). The expression of regulatory genes (*Lag3, Tigit, Ctla4, Pdcd1, Csf1*) was also more evident on day 8 than day 3 samples (Supplementary Fig. S5b) Furthermore, *IL-10* gene expression was observed in the Tr1-, Treg- and, less expected, in the CTL-clusters (Fig. 3e). Thus, the response to infection in lung M2e-specific Trm cells was strikingly enriched for Th17 cell functions and the transcriptome profiles indicated significant functional diversity, including cytotoxicity as well as immune regulation.

### Unexpected functional diversity at the TCR clonotype level of Th17 Trm cells in the lung

**Fig. 4:**
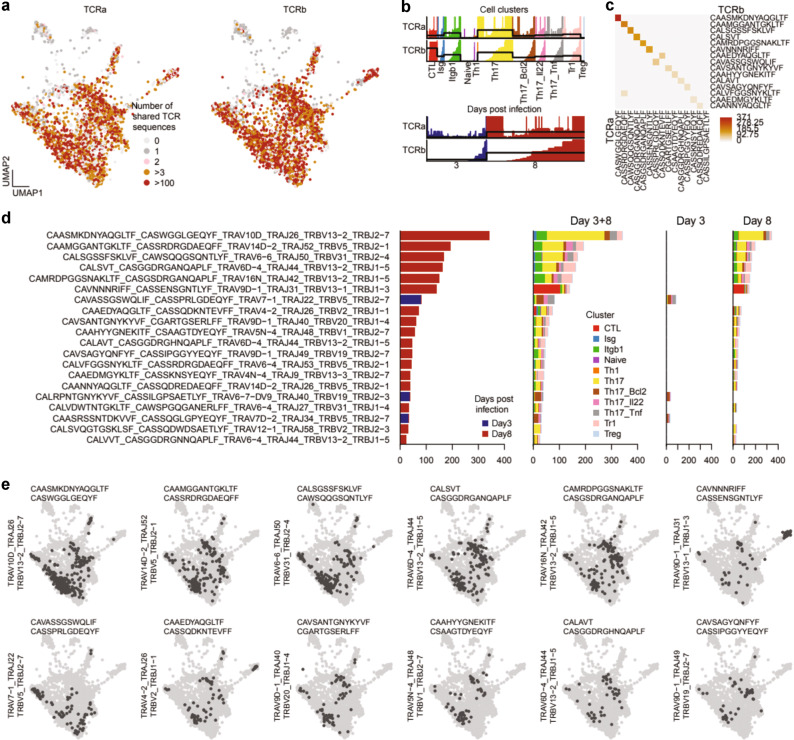
TCR clonal analysis of VDJ gene sequences encoding M2e-specificity in lung Trm cells. Lung M2e-tetramer binding Trm cells from 5 mice were sampled on days 3 or 8 after X47 influenza virus inoculation, and sorted and pooled for 10X for scRNAseq analysis. **a** Distribution of significantly expanded TCR αβ clonotypes (clone size > 20 cells) as found in the UMAP cluster distribution of different M2e-binding Trm cells from Fig. 3c. **b** Cluster distribution of the M2e-binding TCR αβ clonotypes in different functional clusters from day 3 and 8, respectively. **c** Heatmap of the 15 most abundant TCR α and β CDR3-regions in expanded M2e-binding Trm cells. **d** Distribution of the 20 most frequent TCR αβ clonotypes in samples from day 3 or 8 post inoculation (left panel) and the distribution of these into the different functional cluster subsets (right 3 panels). **e** Examples of dominant M2e-specific TCR αβ clonotypes and their representation in the different functional CD4 T cell cluster subsets, according to the UMAP in Fig. 3c.

Next we analyzed which TCR clonotypes that were represented in the responding M2e-specific Trm cells in immunized and challenged mice. The unique gene sequences of the TCRα and β chains were obtained from pooled mice on days 3 and 8 and projected onto the UMAP embedding (Figs. 3c, 4a).^11^ We observed that the naïve-, Treg-, and Th1- clusters were mostly made up of unexpanded clones corresponding to less than 3 cells, likely indicating contaminants or non-responsive clones (Fig. 4a,b). The diversity of TCR CDR3 motifs that could bind to the M2e-tetramer was clearly illustrated by the 15 most abundant combinations of TCRα and β chains that we observed (Fig. 4c). In most cases the defined amino acid CDR3-region of both α and β chains was restricted to one nucleotide sequence (Supplementary Figs. S6, S7). The most highly expanded TCR clonotypes were detected in the Th17, ThCTL, Itgb1 and Isg clusters (Fig. 4a, b). When the distribution of the top 20 most abundant TCR clonotypes with a unique CDR3-region were depicted in day 3 and 8 samples we found three on day 3, while a majority of significantly expanded clonotypes were observed in day 8 samples (Fig. 4d). Most importantly, we observed that the unique clonotypes were represented in several of the clusters, suggesting great functional diversity of Trm cells at the clonal level (Fig. 4d, e, Supplementary Fig. S7a, b). Thus, from the TCR clonotype analysis (VDJ-analysis) we could conclude that individual expanded clones were distributed to different functional clusters with, for example, the same clone hosting cells belonging to the Th17 - and Isg-clusters as well as the Tr1- and/or the ThCTL clusters (Fig. 4d, e, Supplementary Fig. S7a, b). Indeed, the relative distribution of ThCTL and/or Tr1 cells among Th17 clonotypes varied greatly from no to nearly 100%, suggesting that lung Trm cells could give rise to a highly varied progeny of functional subsets, perhaps depending on local conditions upon activation (Supplementary Fig. S7b).

### Lung M2e-specific Th17 Trm cells become increasingly more regulatory in the recovery phase of infection

**Fig. 5:**
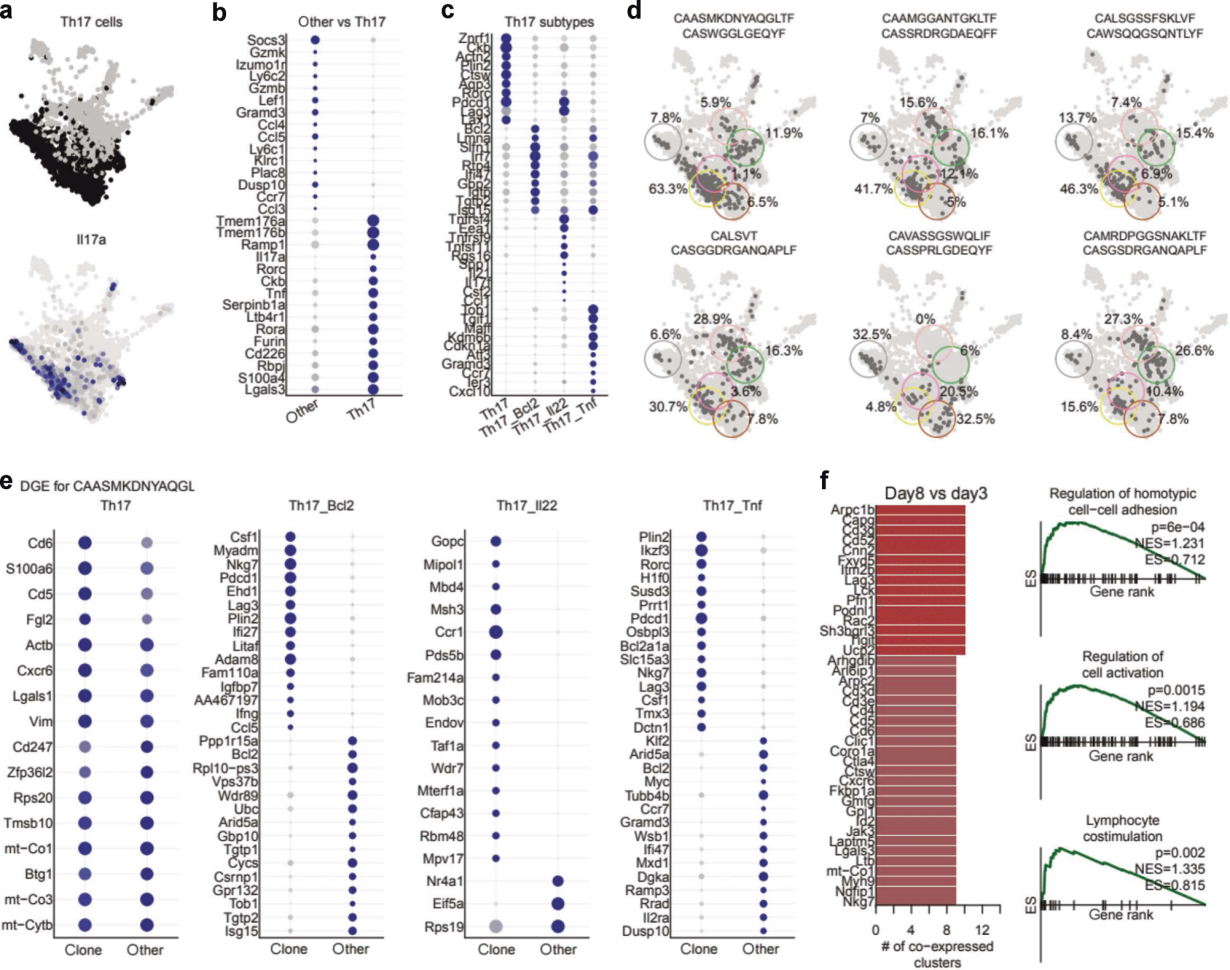
A detailed analysis of the expanded Th17 lineage M2e-specific Trm cells in response to infection. **a** The UMAP cluster visualization of Th17 Trm cells and cells exhibiting IL-17A gene expression. **b** Differentially regulated genes in the four Th17-lineage clusters compared to other M2e-specific Trm cells in non-Th17 clusters. **c** M2e-specific Trm cells in the four different Th17 subclusters also show differential gene expression profiles. **d** Some selected M2e-specific TCR clonotypes and their distribution into different functional clusters and the frequency that they represent in each cluster (circled). **e** Differential gene expression profiles when comparing the CAASMKDNYAQGLTF-CASWGGLGEQYF TCR clonotype with other M2e-specific Trm cells within each of the four Th17 cluster. **f** Bar plot showing the number of genes co-differentially expressed across all M2e-specific CD4 T cell clusters between days 3 and day 8 of infection (left) and their respective gene set enrichment analysis (GSEA) using gene ontology (GO) biological processes (right). The position of the genes in the ranked list are denoted with ticks in the *x*-axis. *P* = *p*-value; ES = enrichment score; NES = normalized enrichment score. Darker shades on the dot plots indicate higher expression. Circle sizes indicate the percentage of cells expressing a gene in a particular cluster.

We further characterized the functional diversity of the four highly abundant *Il17a* gene expressing clusters (Fig. 5a, b). These Th17-lineage related clusters shared expression of *Il17a*, *Rorc*, *Rora*, *Lgals3*, *S100a4*, *Serpinb1a*, *Tmem176a/b, and furin* genes, but were different in the expression of other genes (Fig. 3b, e, S3c, d, 5a–c, and Data S4). Indeed, in a majority of cases we observed M2e-specific TCR clonotypes that were present in all four Th17- clusters, as well as in the related Isg-, Itgb1- and Tr1 clusters, attesting to a high degree of functional diversity of lung M2e-specific Trm cells (Fig. 5c, d). However, with certain other clonotypes we found predominantly archetypical Th17 or ThCTL cells (Fig. 5d, Supplementary Fig. S8). Thus, for example, the most frequent CAASMKDNYAQGLTF-CASWGGLGEQYF Trm clonotype was well represented in the four Th17-lineage clusters as well as in the Tr1, Itgb1, and Isg clusters, but no ThCTLs (Fig. 5e, Supplementary Figs. S7b, S8). On the other hand, the activated M2e-specific ThCTL CAVNNNRIFF_CASSENSGNTLYF clonotype also hosted progeny that expressed Th17-lineage genes (Fig. 4e, Supplementary Fig. 7a, b). An extended analysis of DEG between the CAASMKDNYAQGLTF_CASWGGLGEQYF clonotype and other M2e-specific Trm cells in the respective clusters confirmed that these cells were primarily archetypical Th17 cells as few differences were recorded from the Th17 cluster (Fig. 5e). By inference, more differences were found when analyzing DEG for this clonotype within the Th17:Bcl2,:Tnf or:IL-22 clusters. Interestingly, within each cluster the TCR clonotype demonstrated a shift towards a regulatory profile expressing *Lag3*, *Csf1*, and *Tigit* (Fig. 5e). When comparing the eight most abundant TCR clonotypes across the four different Th17 clusters, we observed that the clonotype impacted greatly on the gene expression profile (Supplementary Fig. S8). Hence, a DEG analysis identified gene profiles that were different for different clonotypes within the same cluster. Finally, a Gene set enrichment analysis (GSEA) of the co-differentially expressed genes between day 3 and 8, using gene ontology (GO) biological processes, confirmed an up-regulation of regulatory genes, including *Cxcr6, Rbpj, Fnbp1, Lrr58*, *Eif3f*, *Ctla4, Lag3, Tigit, Pdcd1, Tgfbr1, IL-10*, and *Smad3* genes, in the day 8 recovery phase samples (Fig. 5f, Supplementary Fig. S4d,n, S5b). Thus, the response to infection in the lung M2e-specific Trm cells showed a clear dominance of Th17-lineage related genes and the clonotypic analysis indicated major functional diversity, including cytotoxicity (ThCTL) and immune regulation (Tr1 cells) in activated and expanded lung Trm cells. Hence, it appears that Th17 Trm cells following i.n immunization with CTA1-3M2e-DD provide a broad range of effector and recovery phase functions, indicative of strong anti-viral control of infection concomitant with effective protection against lung tissue damage.

## Discussion

For broadly protective vaccines against influenza virus infections the ability to stimulate Trm cells in the lung appears critically important.^7,10^ The present study offers valuable insights into the contribution of lung Th17 Trm cells to protection following i.n immunization using the CTA1-3M2e-DD fusion protein.^16^ Although most reports have documented that protection against influenza virus infection is mediated by Th1 effector cells a few studies, including our own, have documented that Th17 cells could be instrumental.^5,51,53,70^ For example, it was observed that IL-10- or Tbet-deficient mice hosted enriched Th17 cells in the lungs, which provided unperturbed protection against infection.^51,53,70^ These reports indicated that specific Th17 cells could protect against influenza virus infections, but the mechanisms responsible for this effect were incompletely investigated, albeit INFγ−production by Th17 cells in *Tbx21*-deficient mice appeared decisive for protection.^20,51^ We have previously documented that IL-17-deficient mice exhibit a 50% reduction in survival from a lethal virus challenge infection following i.n immunizations with CTA1-3M2e-DD as compared to that found in immunized WT mice.^5^ Here we have extended our investigations by using whole and scRNAseq analysis to document the dominance of Th17 Trm cells in the lungs following i.n immunizations. In agreement with other studies, we found that lung Trm cells were perfectly able to expand locally in response to infection without the need to recruit peripheral M2e-specific effector or central memory CD4 T cells, as assessed in our FTY720-treatment experiments.^10,53^ In the *Tbx21*-deficient mouse model anti-IL-17 mAb treatment failed to reduce the level of protection, hence, arguing for an important role of IFNγ.^51^ However, we did not find significant *Tbx21* or *Ifnγ* gene expression in expanded M2e-specific Th17 Trm cells upon infection, (scRNAseq data). Neither did resting M2e-specific lung Trm cells after i.n immunization exhibit gene transcripts (*Tbx21*, *IFNγ*) consistent with Th1 cells. In addition, in vitro recall responses to M2e-peptide were completely dominated by IL-17 production in isolated lung Trm cells. Therefore, we have little reason to think that Th1 cells were involved in protection following i.n immunizations with CTA1-3M2e-DD.

Our TCR-clonotypic analysis of M2e-tetramer binding Trm cells revealed that the expanded clonotypes in the lung harbored not only different functional types of Th17 cells, but also other subsets, such as ThCTL and Tr1 cells. These functional subsets likely contributed to protection and survival in the immunized mice because elimination of CD4 T cells prior to infection resulted in increased viral load and reduced survival. Given the many possible functions of Th17 Trms, documented by the scRNAseq data in the present study, we think complementing mechanisms of protection exist and that IL-17 is just one of several factors that are involved in anti-viral lung protection.^5^ The fact that CD4 T cells can acquire ThCTL functions, restricted by MHC class II-expression has been known for long.^71^ In the context of influenza virus infections CD4 T cells with ThCTL functions have been described in several studies.^32,51,72^ These cells were thought to be derived from Th1 effector cells. However, because our scRNAseq cluster analysis failed to detect expanding cells in the Th1 cluster and cells with a ThCTL gene expression profile shared unique clonotypes with Th17-lineage cells we propose that these cells may have emanated from Th17 Trm cells. To our knowledge this is the first study to indicate that Th17 Trm cells can develop into ThCTL cells. Noteworthy, these cells had a higher expression of the *Tbx21* gene compared to cells in the Th17-lineage clusters, but significantly lower than unexpanded Th1 cells. Virus-specific ThCTLs have been ascribed a protective role because virus-infected epithelial cells express MHC class II molecules and could, thus, be eliminated by ThCTLs, although direct evidence for this in vivo is still missing.^3,34,35,73,74^ We demonstrated that CTA1-3M2e-DD immunized mice hosted CD4 T cells that were cytotoxic to B cells carrying M2e-peptide/MHC II complexes, providing in vitro evidence of a cytotoxic function in M2e-specific Trm cells. Thus, the ThCTLs could help in limiting viral propagation in the lungs as we observed between days 2-4 of infection.

The scRNAseq analysis of M2e-tetramer binding Trm cells revealed a transition from an inflammatory to an anti-inflammatory function in the recovery phase (day 8) of infection, supported also by results from the GSEA. This way the effector stage of infection exhibited pro-inflammatory Th17 functions, while the recovery phase hosted regulatory Th17-cells that contributed to dampening inflammation (Th17) and promoting tissue repair (Th17/IL-22).^75^ Consistent with previous reports that Th17 lineage cells can acquire Tr1 functions we found that the recovery phase hosted archetypical Th17, Th17:IL-22 as well as Tr1 cells with gene expression signatures typical for an anti-inflammatory and immune regulating function.^23,42^ We think this functional breath of expanded lung Th17 Trm cells is important for preventing the extensive lung tissue damage that we have observed in unimmunized control mice following influenza virus infection.^56^ Th17 cells have been described to differentiate into pro-inflammatory as well as anti-inflammatory effector cells depending on the context of activation.^23,41,76,77^ For example, there is considerable knowledge from studying Th17 subsets in the gut intestine that attest to a dynamic functional differentiation of cells from pro-inflammatory to anti-inflammatory functions.^40,41,78^ Although earlier studies have shown that Th17 responses could exacerbate lung pathology upon influenza virus infection,^79^ evidence from *Tbx21*-deficient mice, hosting lungs enriched for Th17 cells, exhibited reduced lung injury and less inflammation in response to infection.^23,53^

The fate of lung CD4 Trm cells upon activation has been inadequately investigated.^31,53^ Current thinking advocates that activated Trm cells largely recall the function of the effector CD4 T cells in the priming immunization.^53,72^ Accordingly a successful influenza vaccine would be expected to generate a range of Trm cells of diverse functions.^3,6,31^ However, the results of the present study somewhat contradicts this notion as we observed that the progeny of the same lung TCR clonotype appeared in effector cells displaying gene expression profiles typifying Th17-, Tr1- as well as ThCTL functions in response to infection. Because a unique TCR clonotype is unlikely to arise from multiple precursors we can assume that the diversity of function that we observed within each M2e-specific Trm clonotype in the lung is a consequence of differentiation upon reactivation rather than subset-specialization following priming.^80^ This notion finds support in that Tr1 or ThCTLs frequencies among the expanded Trm clonotypes varied greatly between 0-100%, relative to the Th17 cell representation, which would be unlikely if the proportion of functional subsets were established already at the priming event (Supplementary Fig. S7). Therefore, we propose that effector cells arising from activation of lung Th17 Trm cells show functional diversity as a reflection of the conditions at the site of activation in the lung. We believe that local cues and the tissue-specific microenvironment can influence differentiation and the type of function required by activated Th17 Trm cells to control infection or prevent tissue damage.^47^ To ultimately answer the question whether differentiation diversity of responding lung Trm cells is the mechanism behind the multiple functional subsets observed within each clonotype or a function of predetermined subset distribution will require lineage-tracing experiments.^81^ However, to the best of our knowledge such experiments cannot be done at present as labeling of lung Trm cells prior to the challenge infection is not technically possible.

## Materials and methods

### Mice and immunizations

Age- and sex-matched BALB/c mice were obtained from Janvier Laboratories (France), while breeding of IgA^−/−^ mice^58^ was done at the Laboratory for Experimental Biomedicine (EBM) (University of Gothenburg, Sweden) under specific pathogen-free conditions. Experiments were carried out at EBM and ethically approved by regional ethical committee. Three immunizations with 5 μg CTA1-3M2e-DD in sterile PBS via the i.n. (20 μL), i.p. (200 μL) or s.c. (200 μL) routes were done with 10 days between the doses.^5^ Control mice received 5 μg CTA1-DD i.n (20 μL) or PBS. Mice were rested 3 weeks or 2 or 6 months after the final i.n immunization on day 20 and sacrificed 3, 4, 6, 8, 10, or 14 days after an influenza A virus challenge infection, as indicated. Serum and lungs were extracted and collected. Serum preparations were stored at −20 ° C until further analysis. For CD4 or CD8 T cell-depletion experiments mice were given 400 µg anti-CD4 mAb (clone GK1.5, BioXCell BE0003-1) or anti-CD8α (clone 2.43 BioXcell) i.p. in 200 μL sterile PBS on 3 consecutive days. Depletion of lung CD4 or CD8 T cells was verified using FACS with a fluorochrome-conjugated, non-competing CD4 mAb (clone RMA-4) or CD8α mAb. To block lymphocyte egress from secondary lymphnodes and spleen mice were given 100 µg FTY720 (Cayman 10006292-50) i.p. in 200 μL sterile PBS every 48 h during the experiment. For testing of Trm status of the M2e-tetramer binding CD4 T cells in the lung, we used the protocol by Anderson et al using i.v injections at 5 min prior to sacrifice of 5 μg of fluorochrome-labeled anti-CD45.1 AF700 mAb (clone A20, BioLegend, #110724).^61^

### Fusion protein

The CTA1-3M2e-DD or CTA1-DD fusion proteins were expressed in *E.coli* and purified by MIVAC Development AB, Sweden, as described in detail.^59^ The purified fusion proteins contained no endotoxin (<1EU/mg of protein), and they bound avidly to IgG in solid phase. The CTA1 moiety demonstrated enzymatic ADP-ribosyltransferase activity as determined by the NAD: agmatine assay.

### Influenza virus challenge

Influenza A virus challenge experiments were performed using a lethal dose of 4x LD50 of the mouse-adapted X47 (H3N2) virus strain (a reassortant between A/Victoria/3/75 (H3N2) and A/Puerto Rico/8/34 (H1N1)) administrated i.n. to mice under light anesthesia. Weight loss and mortality was monitored on a daily basis for two weeks. According to the ethical permit, mice were sacrificed when a loss of body weight exceeded 30% of total body weight. Virus titers were determined by performing a plaque assay in Madin-Darby Canine Kidney (MDCK) cells at a density of 1.5 × 10^5^ per well in 24-well trays (Szretter KJ Curr Protoc Microbiol. 2006 with modifications). Plaque forming units (PFU) were assessed on day 4 after virus inoculation. Briefly, the whole lungs were harvested and homogenized in gentle MACS M tubes containing 2 ml of serum-free DMEM media. The homogenates were then clarified by pelleting the debris and then stored at −80 °C prior to analysis. Upon analysis lung homogenates at 10-fold serial dilutions were incubated with monolayers of MDCK cells for 1 hour at 37 °C followed by washing and then PFUs were counted after an additional 4 days of incubation.

### ELISA and ELISPOT

Anti-M2e antibodies were assessed in serum collected from individual mice at indicated time points. Antibody determinations were performed by ELISA using microtiter plates (MaxiSorp, Nunc) coated with 100 μl of 2.5 μg/ml M2e peptide in 50 mM PBS, pH 9.7 and incubated overnight at +4 °C. After blocking with PBS with 0.1% BSA, serial 1:3 dilutions of the samples were performed starting with 1/25–1/50 dilution. Bound antibodies were detected with alkaline phosphatase-conjugated antibodies directed against mouse IgG, IgG1 or IgG2a diluted 1/1000 (Southern Biotechnology, Birmingham, AL) and after incubation followed by nitrophenyl (NPP) phosphatase substrate (1 mg/ml, SIGMA) in ethanolamine buffer, pH 9.8. Absorbance was measured at 405 nm using a Multiscan MS spectrophotometer. The linear part of the curve was used for calculating titers at a cut-off level of 0.4 above background. Values are given as mean log10 titers ± SD. The ELISPOT assay was used to determine IL-17 or IFNγ producing cells from isolated mLN or lung lymphocytes. The dual kit for IL-17 and IFNγ spot forming cell (SFC) assessments was used according to the manufacturer’s instructions (Immuno Spot, CTL). Briefly, single-cell suspensions of mononuclear cells were seeded in 96-well plates at 1 × 10^5^ cells/well, and 1 μM of peptide was added and the plates were incubated for 24 h (5% CO_2_, 37 °C). Cytokine ELISPOTs were evaluated using a CTL ImmunoSpot analyzer.

### Preparation of lung cells

Single cell suspensions of lung lymphocytes were prepared with the lung dissociation kit from Miltenyi Biotec (ref 130-095-927). Briefly, the harvested lungs were first added into the gentleMACS C tubes containing enzyme mix and broken up into smaller pieces with gentle MAC OctoDissociator (lung 01 program). The samples were then incubated at 37 °C for 30 min before the complete digestion on gentle MAC OctoDissociator (lung 02 program), after which, the cell suspensions were washed once with PBS, passed through a mesh and resuspended in appropriate media for further processing.

### FACS and cell sorting

We assessed the CD4^**+**^ T cell response after immunizations by flow cytometry using the PE-labeled M2e-tetramer, designed and produced by the NIH Tetramer Core Facility (Bethesda, USA) as described.^82^ Tetramer staining was performed at 37 °C for 30 min, with all other fluorochromes and dyes at 4 °C for 30 min. Cells were analyzed using the BD LSR Fortessa X20 and sorted using BD FACSAria III or Aria Fusion (BD Biosciences, USA) using the 85 µm nozzle and analyzed using FlowJo software (Tree Star). Single-cell sorting was performed on singlets, with 4-way purity at a maximum flow rate of 5000–10,000 cells/s. For analysis of expression of surface markers freshly isolated cells were stained with fluorochrome-conjugated antibodies as follows: To exclude dead cells, 7-Aminoactinomycin D (Sigma Aldrich) or Live/dead Aqua (Molecular Probes, Life Technologies, Thermo Scientific) were used. Viable cells were blocked for non-specific staining with 2.4G2 (anti-Fc-receptor) and stained with the following antibodies; anti-CD4-BV785 (RMA4-5), anti-CD19-PECF594 (1D3), CD8-FITC (53-6.7), anti-CD62L-BV605 (MEL-14), anti-NKG2A/C/E-BV421(20d5), anti-CXCR6-BV421 (SA051D1), anti-Rorγt-PE-CF594b (Q31-378), anti-CD3-APC-Cy7 (17A2), anti-CD69-PECF594 (Q31-378), anti-CD44-AF700 (IM7) obtained from Biolegend, Biosciences or BD.

### Total RNAseq analysis

M2e^+^TCRβ^+^ T cells were sorted from cell suspensions derived from 5 pooled mice after dissociation of the lung tissue using Lung Dissociation Kit (130-095-927, Miltenyi Biotec) at 6 months after i.n. immunizations and gene expression profiles were compared with those obtained from sorted naïve (CD44^−^ and CD62L^+^) splenic CD4 T cells. The mRNA was extracted from M2e-tetramer binding and naïve CD4 T cells using µMACS RNA Isolation Kits (Miltenyi 130-090-276). cDNA libraries for RNASeq analyses were subsequently done with 200 ng RNA prepared using TruSeq Stranded RNA Preparation Kit with Ribo-Zero Gold rRNA removal kit to deplete abundant rRNA from purified total RNA. DNA probes bind to rRNA targets, which are both enzymatically-digested to produce total RNA free from abundant transcripts before proceeding with cDNA construction. A first-strand cDNA library was built using annealing purified total RNA and with random hexamers, in presence of First strand Act D Mix and Revers transcriptase which prime the sample for cDNA synthesis (25 °C for 10 min, 42 °C for 15 min, 70 °C for 15 min) and a second cDNA library following by Second Strand cDNA synthesis to generate blunt-ended, double-stranded cDNA fragments. dsDNA Adenylated in 3ʹ ends by adding an adenine (A) nucleotide to the 3ʹ ends of the blunt fragments to prevent them from ligating to each other. Subsequently an anchor ligated to double-stranded cDNA fragments to prepare them for dual indexing. Finally, magnetic beads were used to purify the adapter-ligated fragments for sequencing on an Illumina Nextseq 500 (High Output) to target a minimum of 15 million reads per sample. Whole lung tissue biopsies were prepared on different days following infection as indicated and submerged in 350 µl RLT buffer (Qiagen, Hilden, Germany). The tissue was disrupted and homogenized and RNA extracted using RNeasy mini or micro kits (Qiagen), genomic DNA was eliminated using a DNase treatment step according to the manufacturer’s instructions.

### Single-cell RNA- seq and VDJ-seq analysis

#### Sample preparation

Sorted lung M2e-teramer^+^ TCRβ^+^ T cells (CD19^−^, CD8^−^ F4/80^−^) from 5 mice at each time point were directly loaded onto a Chromium chip (10X Genomics) and run on the Chromium Controller for droplet generation. Reverse transcription was conducted in droplets and cDNA recovered through demulsification and bead purification. Pre-amplified cDNA further went through 10X library preparation for quantification of both mRNA and VDJ sequences {10.1038/nprot.2018.021} using 10X genomics v2 Chemistry kit.

Libraries were sequenced on an Illumina Nextseq500 (High Output) targeting a minimum depth of 10,000 reads per cell across all samples.

#### Identification of cell types

Both single cell RNA-seq and VDJ-seq Reads were pre-processed with 10X Cell Ranger software v.2 and further analyzed in R using mainly Seurat package {10.1038/nbt.4096}. Briefly, UMI counts were summed to the gene level for analysis. Genes detected in less than 3 cells were considered poorly expressed and removed from the analysis. The analysis was mainly focused on protein-coding genes. Cells with total UMI and gene counts above 3 standard deviations from the mean or with less than 200 genes were considered outliers and filtered out before further analysis. Effects from sequencing depth, gene counts, cell cycle, mitochondrial and ribosomal genes were regressed out. Since there were significant technical differences between the two samples, the datasets were integrated using the CCA method as implemented in Seurat.^83^ Integrated data was then processed for principal component analysis (PCA, 100 components), where uniform manifold approximation and projection (UMAP)^84^ and the shared nearest neighbor (SNN, *k* = 10) graph were computed. Louvain clustering was performed on the SNN graph at varying resolutions. Differential gene expression was done using Wilcoxon test for each group, where genes with absolute log2 fold change above 0.1 and p-values below 0.05 were considered significant. Differentially expressed genes were further used for gene set enrichment analysis (GSEA)^85^ with the fgsea package {10.1101/060012} using gene ontology (GO) annotations. The comprehensive annotation of cytokines, transcription factors, receptors, and other genes related to the immune response were defined elsewhere {10.1126/sciimmunol.aal2192 }. Pre-filtered abundance tables from Cell Ranger were used for VDJ analysis. Since most cells have different amounts of predicted VDJ sequences, only the most abundant TCR*α* and TCR*β* sequences found within each cell were assigned. For reproducibility purposes, all the analysis steps were done in controlled Conda environments^86^ via the Sauron pipeline (unpublished, https://github.com/NBISweden/sauron). All analysis steps performed in this manuscript from counts to the main figures can be recreated by following the instructions in the Supplementary Data. Filtering, clustering, and differential expression analysis were performed with Seurat (Satija Lab).

### In-vitro ThCTL assay

BALB/c mice were immunized i.n. with 5 µg CTA1-3M2e-DD or PBS on days 0, 10, and 20. Mice were rested for 6 months before infection with 4xLD50 influenza X47 virus. Four days post-infection CD4 T cells were extracted by negative selection from pooled lung tissue and co-cultured at 10^6^ cells and serial dilutions with a 1:1 mix of peptide-pulsed CFSE^lo^ (0.4 μM) and non-pulsed CFSE^hi^ (1.0 μM) spleen-derived target B cells for 6 h at 37 °C before analysis by FACS. B cells were incubated in CFSE for 15 min at 37 °C before washing. CFSE^+^ B cells were pulsed with M2e-peptide (5 μg/mL, Innovagen AB) for 1 h at 37 °C. CD4 isolation kit (STEMCELL #19852) and B cell isolation kit (STEMCELL #19844).

### Statistics

All statistical analyses were performed with Prism 7 for Mac OS (GraphPad Software, Inc.).

Mean ± SD is depicted, unless otherwise stated. Infection groups had 10 mice in each group and immunoassessments had 5 mice in each group. Reproducibility was secured by 2-4 experiments in each category. Log-rank test applied for survival comparisons. Experiments were Unpaired, two-tailed *t*-test ± Holm-Sidak correction or One-way ANOVA test with Tukey’s correction applied as indicated. Unpaired, two-tailed *t*-test with Benjamini-Hochberg correction applied for differential gene expression. *p ≤ 0.05, **p ≤ 0.01, ***p ≤ 0.001, ****p ≤ 0.0001 or as stated.

## Supplementary information

Supplementary Figure Legends

Supplementary Figures
