# Supplementary material for: Clonotypic analysis of protective influenza M2e-specific lung resident Th17 memory cells reveals extensive functional diversity
Source: Mucosal Immunol. 2022 Mar 8;15(4):717–29. doi: 10.1038/s41385-022-00497-9 (PMC8903128; doi:10.1038/s41385-022-00497-9)
Supplement: Supplementary file 1 — Supplementary Figure Legends [file 41385_2022_497_MOESM1_ESM.docx]

**Supplementary Figure Legends**

**Figure S1**

**Intranasal immunization with CTA1-3M2e-DD stimulates M2e-specific CD4 T cell resident memory cells in the lung**

(a) In vitro cultures of splenocytes from i.n immunized mice were stimulated with M2e-recall peptide after depletion of CD4 or CD8 T cells. The impact of T cell depletion on M2e T cell proliferation was determined after 72h with 3H-thymidine incorporation for the last 6h and values are given for triplicate cultures in mean cpm ± SD of 3 mice and one representative experiment of two giving similar results is shown. (b) Mice (n=10) immunized i.n as in Fig. 1c developed local IgA antibodies specific for M2e which were detectable in their bronchioalveolar lavage at sacrifice and given in log_10_ titers± SD for indicated groups. (c) The virus titers in lungs of immunized mice (n=6) at 4 days post challange after CD4 or CD8 T cell depletion in vivo, as indicated and values are given as pfu/lung ± SD for each group. (d) Corresponding serum anti-M2e IgG2a log_10_ titers± SD to the total IgG titers given for i.n immunized mice prior to challenge infection. (e) Evidence that AP7 conjugated anti-CD45.1 mAb given i.v 5 min prior to sacrifice does not bind to M2e-tetramer specific cells by FACS. Furthermore, anti-CD44 and CD4 identifies resident memory CD4 T cells in the lungs while no cells are detectable in the blood (right panel).

**Figure S2.**

**Single cell RNA-seq quality metrics and UMAP clustering**.(a) Expression of T cell marker genes used to sort cells Cd3e, Cd3g, Thy1 and Cd4. Cd8a and Cd8b gene expression was not found in the dataset. (b) SNN graph overlay onto the UMAP embedding. Each line represents a connection between 2 cells. Cells are coloured by the clusters identified with Louvain. For practical reasons, only 30% of the lines are visualized. A graph abstraction with black lines for each cluster is overlaid on top of the UMAP. Lines represent that >2% of the cluster cells are connected and the thickness indicates the percentage of connections between 2 clusters. (c) Quality metrics visualized as bar plots for day 3 and 8 of infection (left) or separated per cell cluster (right). Black lines represent the average values for each group.

**Figure S3.**

**Functional classification of differentially expressed genes across all clusters.** (a) Cytokines (b) Receptors (c) Trascription Factors and (d) other genes. The top 6 genes per clusters are shown, sorted by p-value and log differential detection percentage.

**Figure S4**

**Expression of cluster-defining key marker genes projected onto the UMAP embedding of the M2e-binding CD4 T cells. (**a) Naïve T cells, (b) T follicular helper cells (Tfh), (c) Foxp3-pos regulatory T cells (Treg), (d) Foxp3-neg regulatory T cells (Tr1), (e) T helper 1 cells (Th1), (f) Cytotoxic CD4 T cells (CTL, (g) Cells hosting interferon-stimulated genes (Isg), (h) Integrinbeta-1 transitional cells, (i) Archetypical T helper 17 cells, (j) Classical T helper 17 cells, (k) IL22^+^ T helper 17 cells (Th17:IL-22 or Th22). (l) Tnf^+^ T helper 17 cells (Th17:Tnf /“pathogenic”"Th17 cells) (m) Bcl2^+^ T helper 17 (Th17:Bcl2) (n) Cells expressing regulatory genes.

**Figure S5.**

**Conserved up- (a) or down-regulated (b) genes in day 3 and day 8 M2e-specific Trm cells**. Asterisks show whether that gene is differentilaly expressed in day 3 relative to day 8 samples (a) or day 8 relative to day 3 samples (b). Bar plots on the right represent how many clusters that the gene was found differentially expressed in.

**Figure S6.**

**Differential VDJ gene usage for TCR-α and TCR-β CDR3-regions in M2e-tetramer binding cells** (a) Heatmap of TCR-α pairings with complete VDJ gene sequences, (b) TCR-α pairings with V-chain sequences only or (c) TCR-α pairings with J-chain sequences only. (d) Heatmap of TCR-β pairings with complete VDJ gene sequences, (e) TCR-β pairings with V-chain sequences only or (f) TCR-β pairings with J-chain sequences only.

**Figure S7.**

**TCR clonotype assessments using VDJ gene usage analysis for unique α and β chain CDR3-regions of M2e-tetramer binding cells**. a) Distribution of the most expanded TCR clonotypes and b) their distribution in day 3 and day 8 samples with individual cells from each clonotype in the different functional subsets.

**Figure S8**

**Differential gene expression of the eight most abundant M2e-specific TCR clonotypes in the four different Th17-subclusters .** The unique clonotypes are listed above each column and the Th17 subclusters are listed in rows a-d.

**Description of supplementary data files**

**Supplementary Data 1.** Table

**Supplementary Data 2.** Table

**Supplementary Data 3.** Table

**Supplementary Data 4.** Table
