# Supplementary material for: Clonotypic analysis of protective influenza M2e-specific lung resident Th17 memory cells reveals extensive functional diversity
Source: Mucosal Immunol. 2022 Mar 8;15(4):717–29. doi: 10.1038/s41385-022-00497-9 (PMC8903128; doi:10.1038/s41385-022-00497-9)
Supplement: Supplementary file 2 — Supplementary Figures [file 41385_2022_497_MOESM2_ESM.pdf]

Figure S1

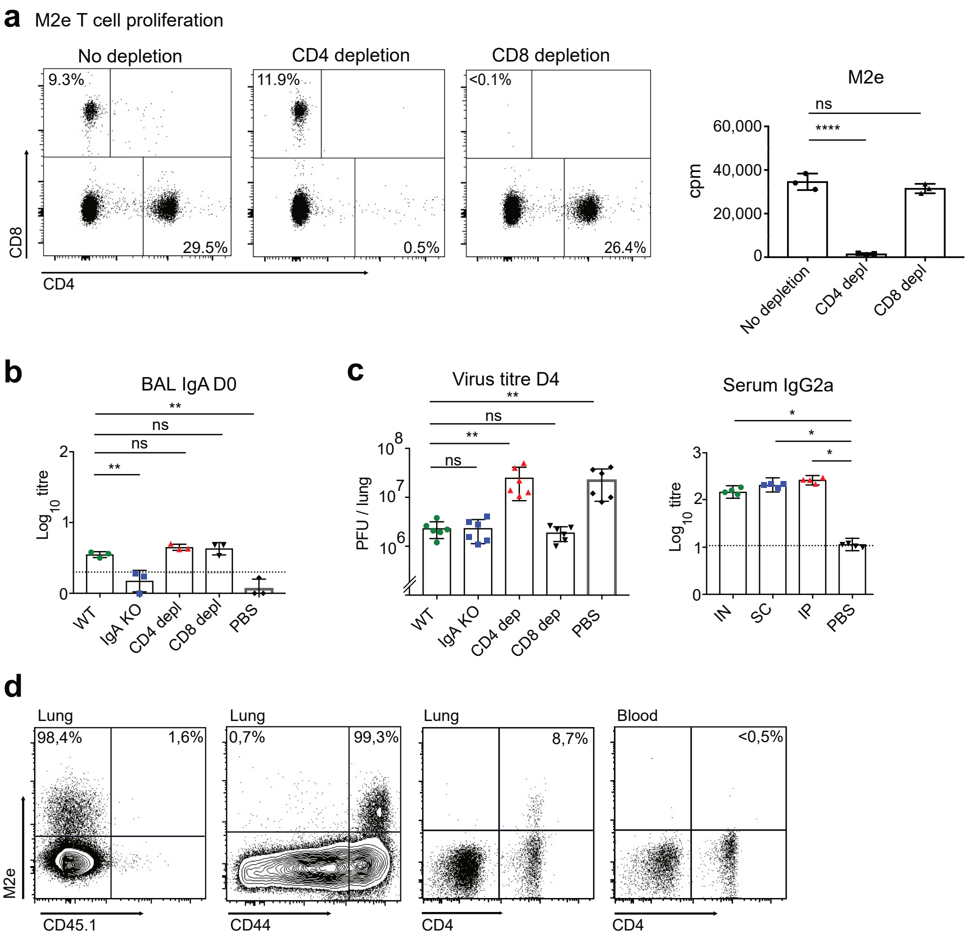
a
M2e T cell proliferation
No depletion
CD4 depletion
CD8 depletion
9.3%
29.5%
11.9%
0.5%
<0.1%
26.4%
CD8
CD4
M2e
ns
****
cpm
60,000
40,000
20,000
0
No depletion
CD4 depl
CD8 depl
b
BAL IgA D0
**
ns
ns
**
Log10 titre
WT
IgA KO
CD4 depl
CD8 depl
PBS
c
Virus titre D4
**
ns
**
ns
PFU / lung
WT
IgA KO
CD4 dep
CD8 dep
PBS
Serum IgG2a
*
*
*
Log10 titre
IN
SC
IP
PBS
d
Lung
98,4%
1,6%
Lung
0,7%
99,3%
Lung
8,7%
Blood
<0,5%
M2e
CD45.1
CD44
CD4
CD4

Figure S2

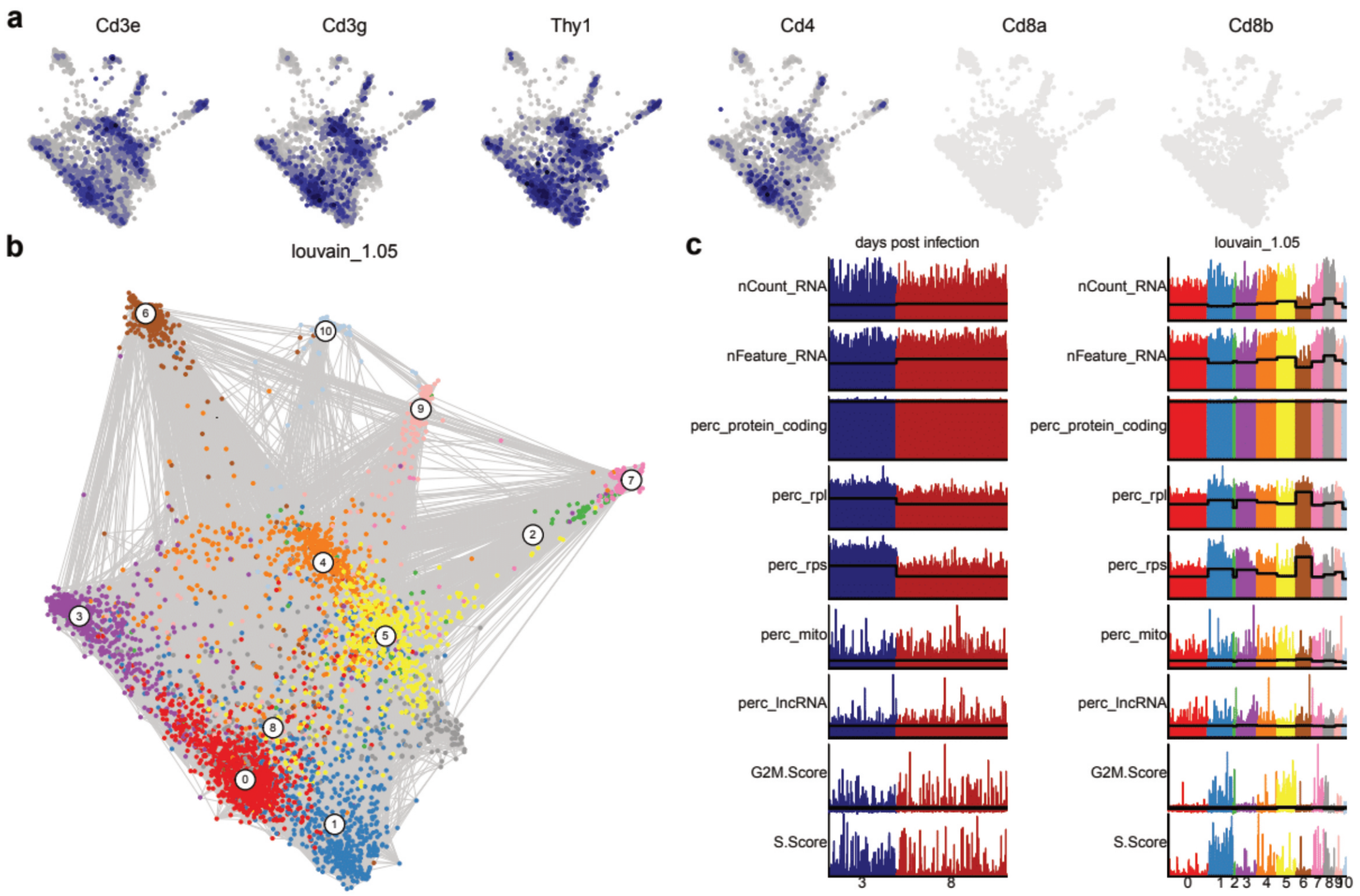
a
Cd3e
Cd3g
Thy1
Cd4
Cd8a
Cd8b
b
louvain_1.05
0
1
2
3
4
5
6
7
8
9
10
c
days post infection
louvain_1.05
nCount_RNA
nFeature_RNA
perc_protein_coding
perc_rpl
perc_rps
perc_mito
perc_lncRNA
G2M.Score
S.Score
3
8
0
1
2
3
4
5
6
7
8
9
10

Figure S3

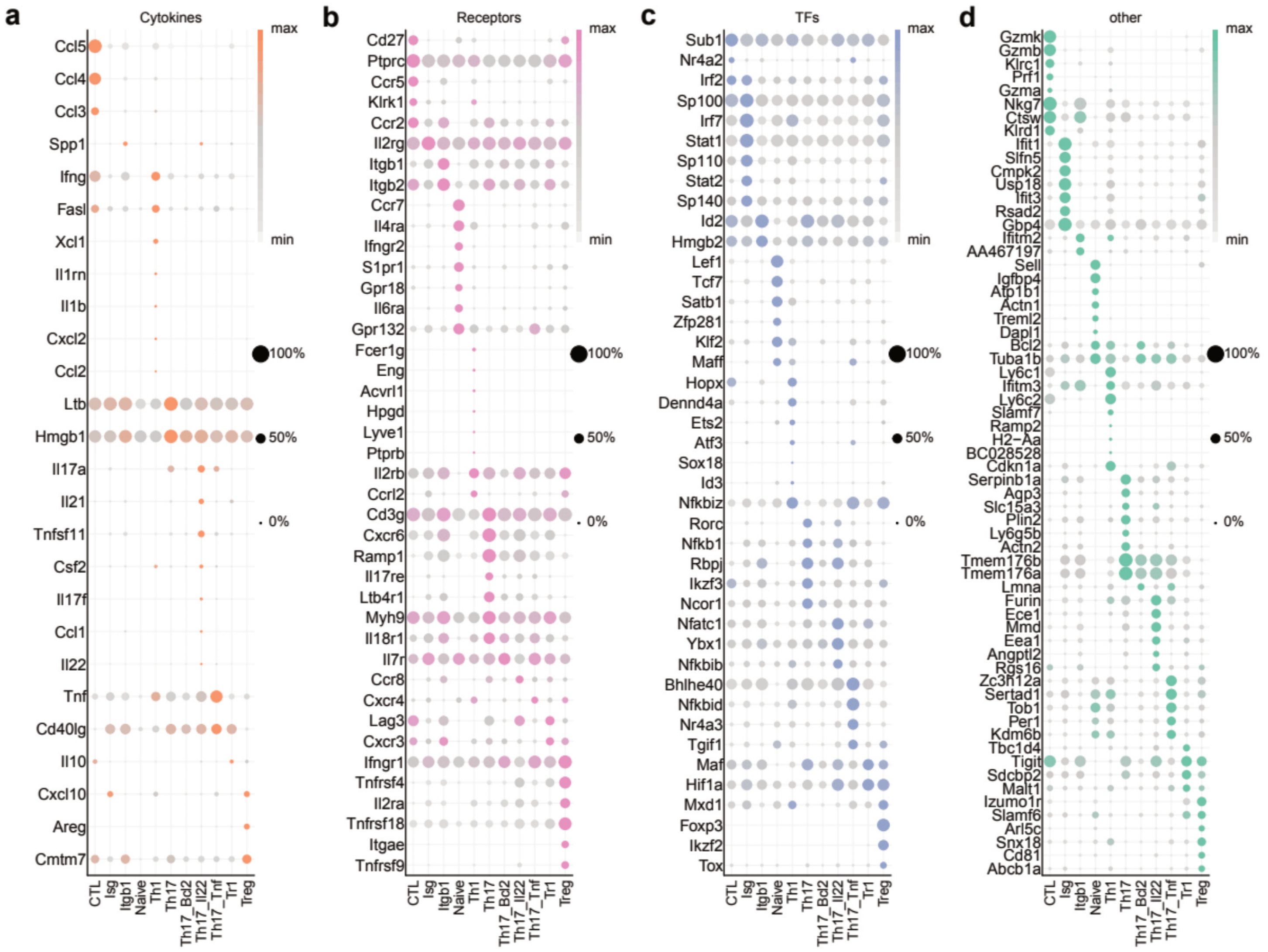
a
Cytokines
b
Receptors
c
TFs
d
other
max
min
100%
50%
0%
CTL
Isg
Itgb1
Naive
Th1
Th17
Th17_Bcl2
Th17_Il22
Th17_Tnf
Tr1
Treg
Ccl5
Ccl4
Ccl3
Spp1
Ifng
Fasl
Xcl1
Il1rn
Il1b
Cxcl2
Ccl2
Ltb
Hmgb1
Il17a
Il21
Tnfsf11
Csf2
Il17f
Ccl1
Il22
Tnf
Cd40lg
Il10
Cxcl10
Areg
Cmtm7
Cd27
Ptprc
Ccr5
Klrk1
Ccr2
Il2rg
Itgb1
Itgb2
Ccr7
Il4ra
Ifngr2
S1pr1
Gpr18
Il6ra
Gpr132
Fcer1g
Eng
Acvrl1
Hpgd
Lyve1
Ptprb
Il2rb
Ccrl2
Cd3g
Cxcr6
Ramp1
Il17re
Ltb4r1
Myh9
Il18r1
Il7r
Ccr8
Cxcr4
Lag3
Cxcr3
Ifngr1
Tnfrsf4
Il2ra
Tnfrsf18
Itgae
Tnfrsf9
Sub1
Nr4a2
Irf2
Sp100
Irf7
Stat1
Sp110
Stat2
Sp140
Id2
Hmgb2
Lef1
Tcf7
Satb1
Zfp281
Klf2
Maff
Hopx
Dennd4a
Ets2
Atf3
Sox18
Id3
Nfkbiz
Rorc
Nfkb1
Rbpj
Ikzf3
Ncor1
Nfatc1
Ybx1
Nfkbib
Bhlhe40
Nfkbid
Nr4a3
Tgif1
Maf
Hif1a
Mxd1
Foxp3
Ikzf2
Tox
Gzmk
Gzmb
Klrc1
Prf1
Gzma
Nkg7
Ctsw
Klrd1
Ifit1
Slfn5
Cmpk2
Usp18
Ifit3
Rsad2
Gbp4
Ifitm2
AA467197
Sell
Igfbp4
Atp1b1
Actn1
Treml2
Dapl1
Bcl2
Tuba1b
Ly6c1
Ifitm3
Ly6c2
Slamf7
Ramp2
H2-Aa
BC028528
Cdkn1a
Serpinb1a
Aqp3
Slc15a3
Plin2
Ly6g5b
Actn2
Tmem176b
Tmem176a
Lmna
Furin
Ece1
Mmd
Eea1
Angptl2
Rgs16
Zc3h12a
Sertad1
Tob1
Per1
Kdm6b
Tbc1d4
Tigit
Sdcbp2
Malt1
Izumo1r
Slamf6
Arl5c
Snx18
Cd81
Abcb1a

Figure S4

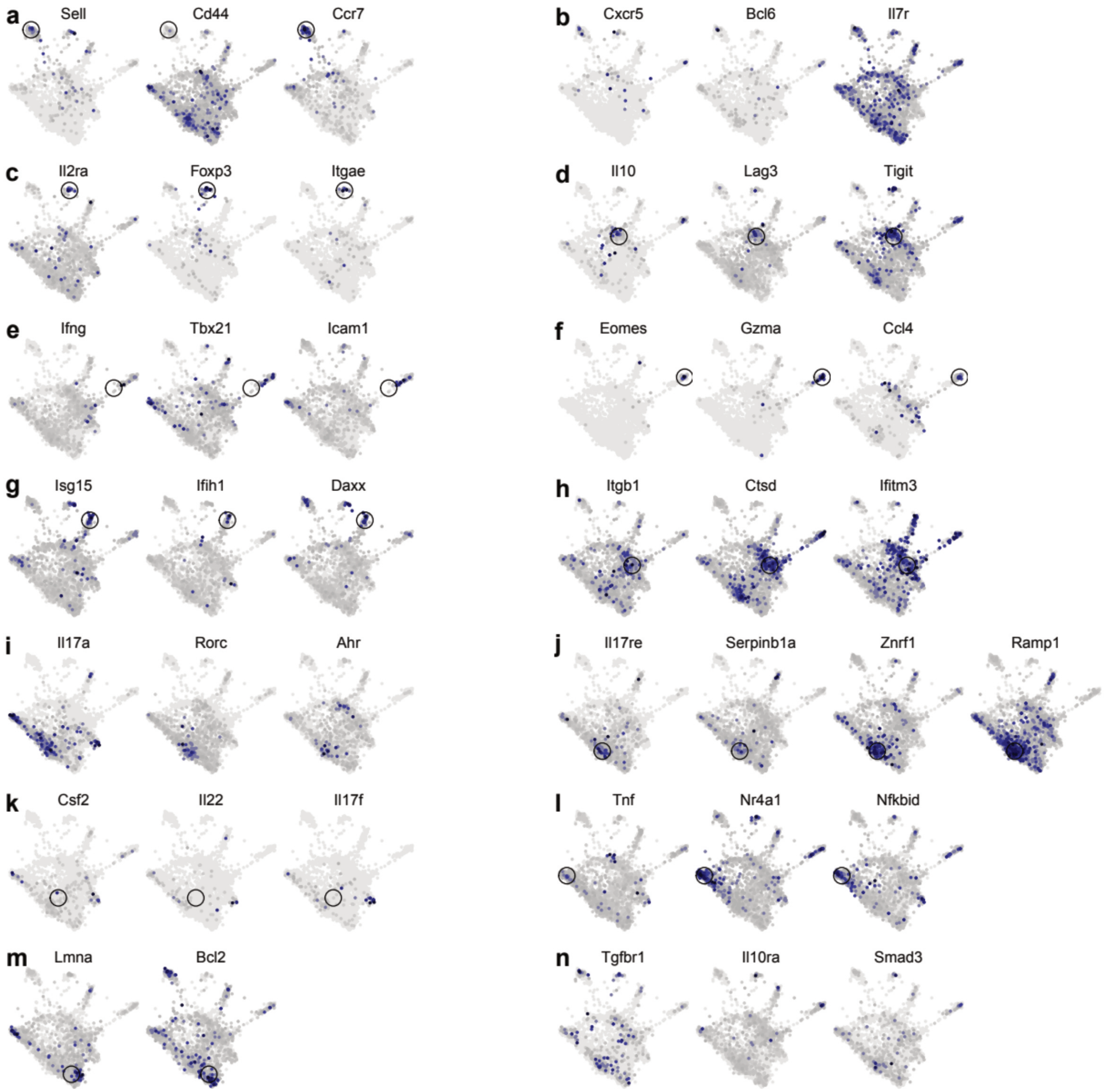
a
Sell
Cd44
Ccr7
b
Cxcr5
Bcl6
Il7r
c
Il2ra
Foxp3
Itgae
d
Il10
Lag3
Tigit
e
Ifng
Tbx21
Icam1
f
Eomes
Gzma
Ccl4
g
Isg15
Ifih1
Daxx
h
Itgb1
Ctsd
Ifitm3
i
Il17a
Rorc
Ahr
j
Il17re
Serpinb1a
Znrf1
Ramp1
k
Csf2
Il22
Il17f
l
Tnf
Nr4a1
Nfkbid
m
Lmna
Bcl2
n
Tgfbr1
Il10ra
Smad3

Figure S5

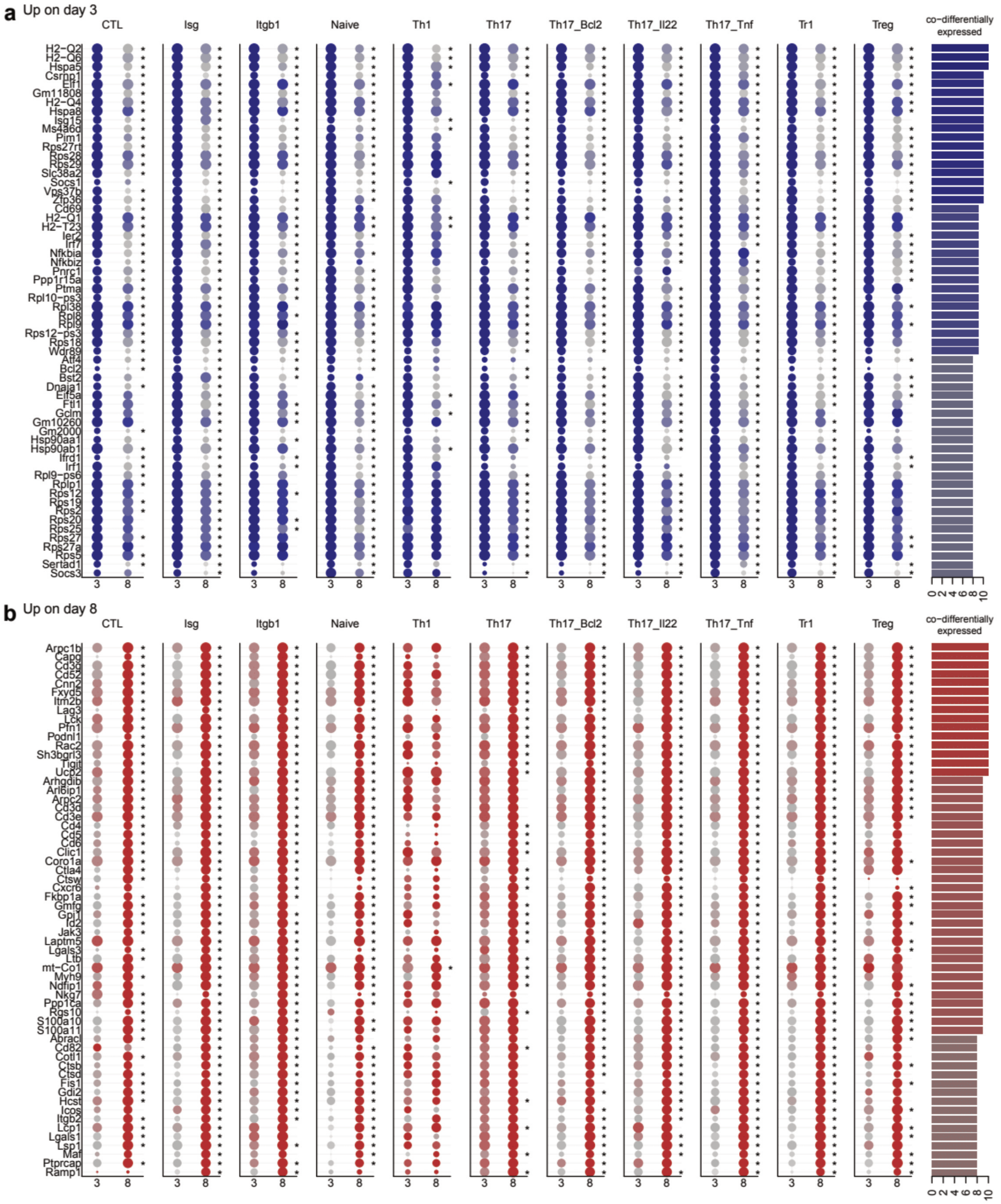
a
Up on day 3
CTL
Isg
Itgb1
Naive
Th1
Th17
Th17_Bcl2
Th17_Il22
Th17_Tnf
Tr1
Treg
co−differentially expressed
H2−Q2
H2−Q6
Hspa5
Csrnp1
Elf1
Gm11808
H2−Q4
Hspa8
Isg15
Ms4a6d
Pim1
Rps27rt
Rps28
Rps29
Slc38a2
Socs1
Vps37b
Zfp36
Cd69
H2−Q1
H2−T23
Ier2
Irf7
Nfkbia
Nfkbiz
Pnrc1
Ppp1r15a
Ptma
Rpl10−ps3
Rpl38
Rpl8
Rpl9
Rps12−ps3
Rps18
Wdr89
Atf4
Bcl2
Bst2
Dnaja1
Eif5a
Ftl1
Gclm
Gm10260
Gm2000
Hsp90aa1
Hsp90ab1
Ifrd1
Irf1
Rpl9−ps6
Rplp1
Rps12
Rps19
Rps2
Rps20
Rps25
Rps27
Rps27a
Rps5
Sertad1
Socs3
3
8
0 2 4 6 8 10
b
Up on day 8
CTL
Isg
Itgb1
Naive
Th1
Th17
Th17_Bcl2
Th17_Il22
Th17_Tnf
Tr1
Treg
co−differentially expressed
Arpc1b
Capg
Cd3g
Cd52
Cnn2
Fxyd5
Itm2b
Lag3
Lck
Pfn1
Podnl1
Rac2
Sh3bgrl3
Tigit
Ucp2
Arhgdib
Arl6ip1
Arpc2
Cd3d
Cd3e
Cd4
Cd5
Cd6
Clic1
Coro1a
Ctla4
Ctsw
Cxcr6
Fkbp1a
Gmfg
Gpi1
Id2
Jak3
Laptm5
Lgals3
Ltb
mt−Co1
Myh9
Ndfip1
Nkg7
Ppp1ca
Rgs10
S100a10
S100a11
Abracl
Cd82
Cotl1
Ctsb
Ctsd
Fis1
Gdi2
Hcst
Icos
Itgb2
Lcp1
Lgals1
Lsp1
Maf
Ptprcap
Ramp1
3
8
0 2 4 6 8 10

Figure S6

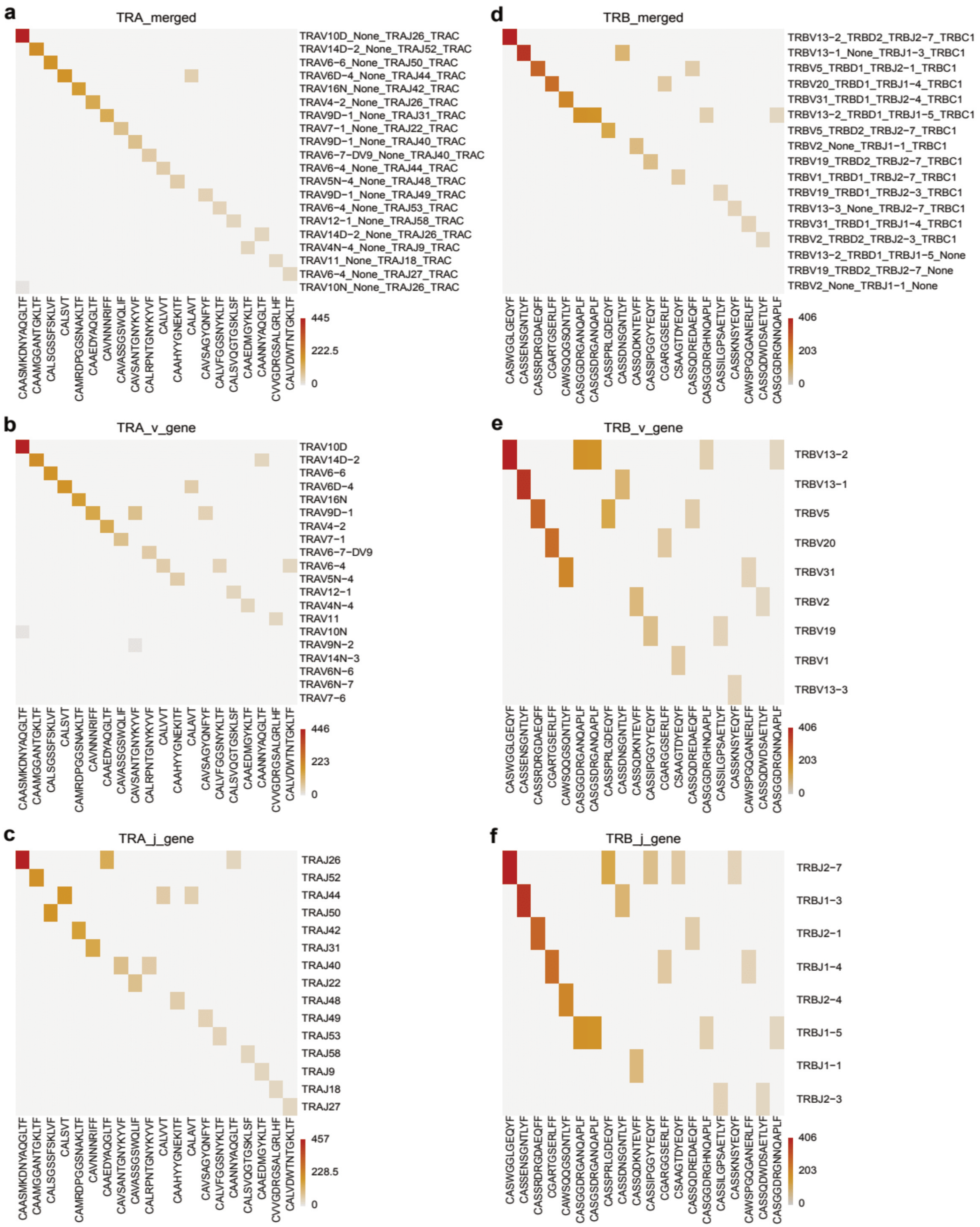
a
TRA_merged
TRAV10D_None_TRAJ26_TRAC
TRAV14D−2_None_TRAJ52_TRAC
TRAV6−6_None_TRAJ50_TRAC
TRAV6D−4_None_TRAJ44_TRAC
TRAV16N_None_TRAJ42_TRAC
TRAV4−2_None_TRAJ26_TRAC
TRAV9D−1_None_TRAJ31_TRAC
TRAV7−1_None_TRAJ22_TRAC
TRAV9D−1_None_TRAJ40_TRAC
TRAV6−7−DV9_None_TRAJ40_TRAC
TRAV6−4_None_TRAJ44_TRAC
TRAV5N−4_None_TRAJ48_TRAC
TRAV9D−1_None_TRAJ49_TRAC
TRAV6−4_None_TRAJ53_TRAC
TRAV12−1_None_TRAJ58_TRAC
TRAV14D−2_None_TRAJ26_TRAC
TRAV4N−4_None_TRAJ9_TRAC
TRAV11_None_TRAJ18_TRAC
TRAV6−4_None_TRAJ27_TRAC
TRAV10N_None_TRAJ26_TRAC
445
222.5
0
b
TRA_v_gene
TRAV10D
TRAV14D−2
TRAV6−6
TRAV6D−4
TRAV16N
TRAV9D−1
TRAV4−2
TRAV7−1
TRAV6−7−DV9
TRAV6−4
TRAV5N−4
TRAV12−1
TRAV4N−4
TRAV11
TRAV10N
TRAV9N−2
TRAV14N−3
TRAV6N−6
TRAV6N−7
TRAV7−6
446
223
0
c
TRA_j_gene
TRAJ26
TRAJ52
TRAJ44
TRAJ50
TRAJ42
TRAJ31
TRAJ40
TRAJ22
TRAJ48
TRAJ49
TRAJ53
TRAJ58
TRAJ9
TRAJ18
TRAJ27
457
228.5
0
d
TRB_merged
TRBV13−2_TRBD2_TRBJ2−7_TRBC1
TRBV13−1_None_TRBJ1−3_TRBC1
TRBV5_TRBD1_TRBJ2−1_TRBC1
TRBV20_TRBD1_TRBJ1−4_TRBC1
TRBV31_TRBD1_TRBJ2−4_TRBC1
TRBV13−2_TRBD1_TRBJ1−5_TRBC1
TRBV5_TRBD2_TRBJ2−7_TRBC1
TRBV2_None_TRBJ1−1_TRBC1
TRBV19_TRBD2_TRBJ2−7_TRBC1
TRBV1_TRBD1_TRBJ2−7_TRBC1
TRBV19_TRBD1_TRBJ2−3_TRBC1
TRBV13−3_None_TRBJ2−7_TRBC1
TRBV31_TRBD1_TRBJ1−4_TRBC1
TRBV2_TRBD2_TRBJ2−3_TRBC1
TRBV13−2_TRBD1_TRBJ1−5_None
TRBV19_TRBD2_TRBJ2−7_None
TRBV2_None_TRBJ1−1_None
406
203
0
e
TRB_v_gene
TRBV13−2
TRBV13−1
TRBV5
TRBV20
TRBV31
TRBV2
TRBV19
TRBV1
TRBV13−3
406
203
0
f
TRB_j_gene
TRBJ2−7
TRBJ1−3
TRBJ2−1
TRBJ1−4
TRBJ2−4
TRBJ1−5
TRBJ1−1
TRBJ2−3
406
203
0

Figure S7

a

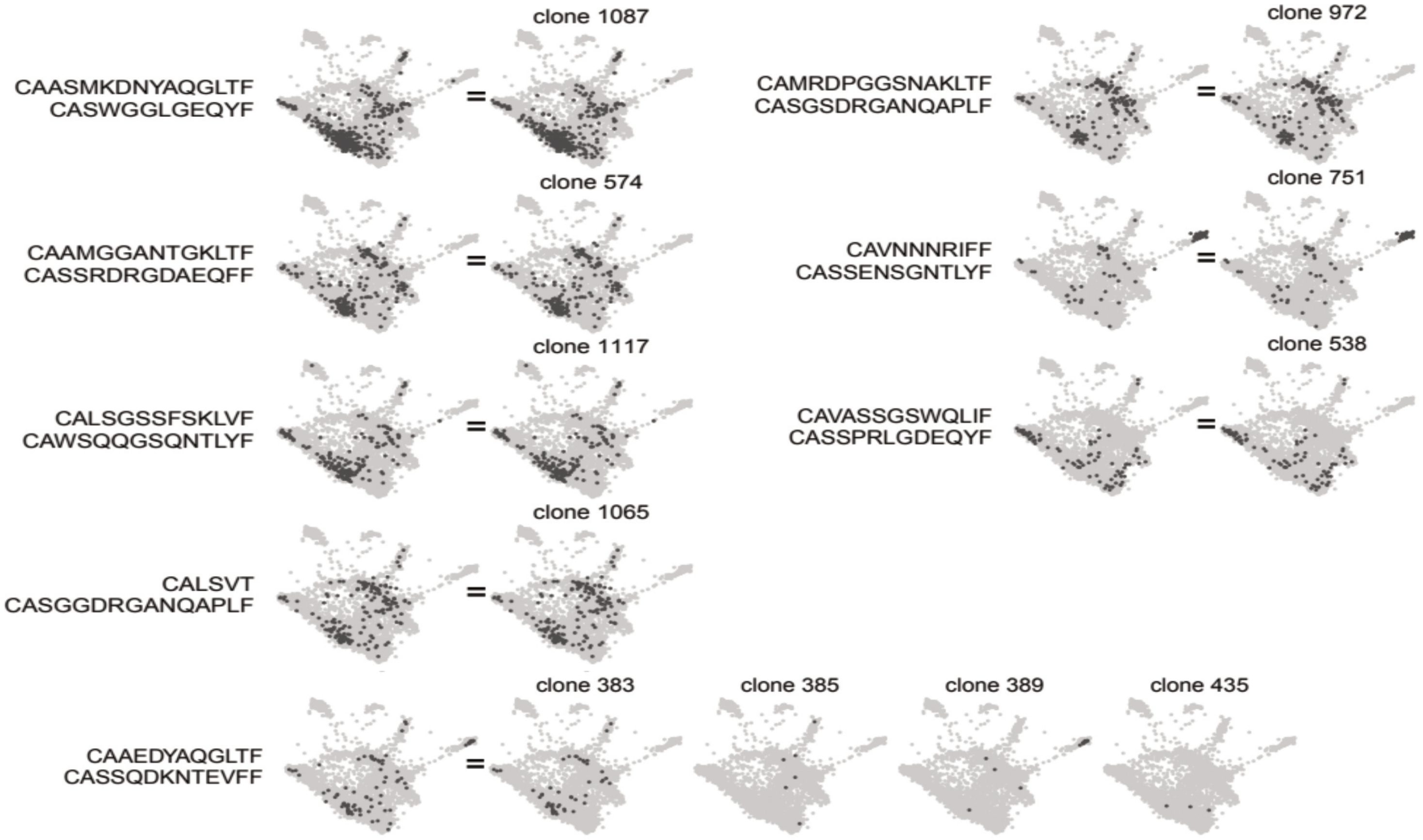

b

	CTL	Isg	Itgb1	Naive	Th1	Th17	Th17_Bcl2	Th17_Il22	Th17_Tnf	Tr1	Treg	
CAVASSGSWQLIF_CASSPRLGDEQYF_TRAV7−1_TRAJ22_TRBV5_TRBJ2−7	0	3	5	0	0	4	27	17	26	0	0	day 3
CALRPNTGNYKYVF_CASSILGPSAETLYF_TRAV6−7−DV9_TRAJ40_TRBV19_TRBJ2−3	0	0	4	1	2	0	24	2	4	1	0	
CAASRSSNTDKVVF_CASSQGLGPYEQYF_TRAV7D−2_TRAJ34_TRBV5_TRBJ2−7	0	0	0	0	0	3	17	6	5	1	0	
CAASMKDNYAQGLTF_CASWGGLGEQYF_TRAV10D_TRAJ26_TRBV13−2_TRBJ2−7	1	11	41	0	0	220	20	2	26	22	1	day 8
CAAMGGANTGKLTF_CASSRDRGDAEQFF_TRAV14D−2_TRAJ52_TRBV5_TRBJ2−1	0	4	31	1	0	81	10	24	14	29	0	
CALSGSSFSKLVF_CAWSQQGSQNTLYF_TRAV6−6_TRAJ50_TRBV31_TRBJ2−4	1	6	27	1	0	78	9	11	23	13	0	
CALSVT_CASGGDRGANQAPLF_TRAV6D−4_TRAJ44_TRBV13−2_TRBJ1−5	0	8	27	2	0	51	12	6	10	47	0	
CAMRDPGGSNAKLTF_CASGSDRGANQAPLF_TRAV16N_TRAJ42_TRBV13−2_TRBJ1−5	0	5	40	1	0	23	10	16	13	42	0	
CAVNNNRIFF_CASSENSGNTLYF_TRAV9D−1_TRAJ31_TRBV13−1_TRBJ1−3	103	1	7	1	0	8	4	1	5	11	0	
CAAEDYAQGLTF_CASSQDKNTEVFF_TRAV4−2_TRAJ26_TRBV2_TRBJ1−1	12	5	12	1	0	19	5	2	6	11	0	
CAVSANTGNYKYVF_CGARTGSERLFF_TRAV9D−1_TRAJ40_TRBV20_TRBJ1−4	0	2	9	1	0	21	6	0	10	12	0	
CAAHYYGNEKITF_CSAAGTDYEQYF_TRAV5N−4_TRAJ48_TRBV1_TRBJ2−7	1	1	8	0	0	22	5	4	4	12	0	
CALAVT_CASGGDRGHNQAPLF_TRAV6D−4_TRAJ44_TRBV13−2_TRBJ1−5	0	0	7	0	0	10	4	3	4	18	0	
CAVSAGYQNFYF_CASSIPGGYYEQYF_TRAV9D−1_TRAJ49_TRBV19_TRBJ2−7	0	1	20	0	0	9	1	7	3	1	4	
CALVFGGSNYKLTF_CASSRDRGDAEQFF_TRAV6−4_TRAJ53_TRBV5_TRBJ2−1	0	5	4	1	0	17	3	6	4	4	0	
CAAEDMGYKLTF_CASSKNSYEQYF_TRAV4N−4_TRAJ9_TRBV13−3_TRBJ2−7	0	1	6	2	0	5	0	3	0	23	0	
CAANNYAQGLTF_CASSQDREDAEQFF_TRAV14D−2_TRAJ26_TRBV5_TRBJ2−1	0	3	7	0	0	18	2	5	2	1	0	
CALVDWTNTGKLTF_CAWSPGQGANERLFF_TRAV6−4_TRAJ27_TRBV31_TRBJ1−4	0	2	7	0	0	12	3	3	4	3	0	
CALSVQGTGSKLSF_CASSQDWDSAETLYF_TRAV12−1_TRAJ58_TRBV2_TRBJ2−3	0	0	3	0	0	24	2	1	0	2	0	
CALVVT_CASGGDRGNNQAPLF_TRAV6−4_TRAJ44_TRBV13−2_TRBJ1−5	0	0	5	1	0	10	1	1	1	6	0	

Figure S8

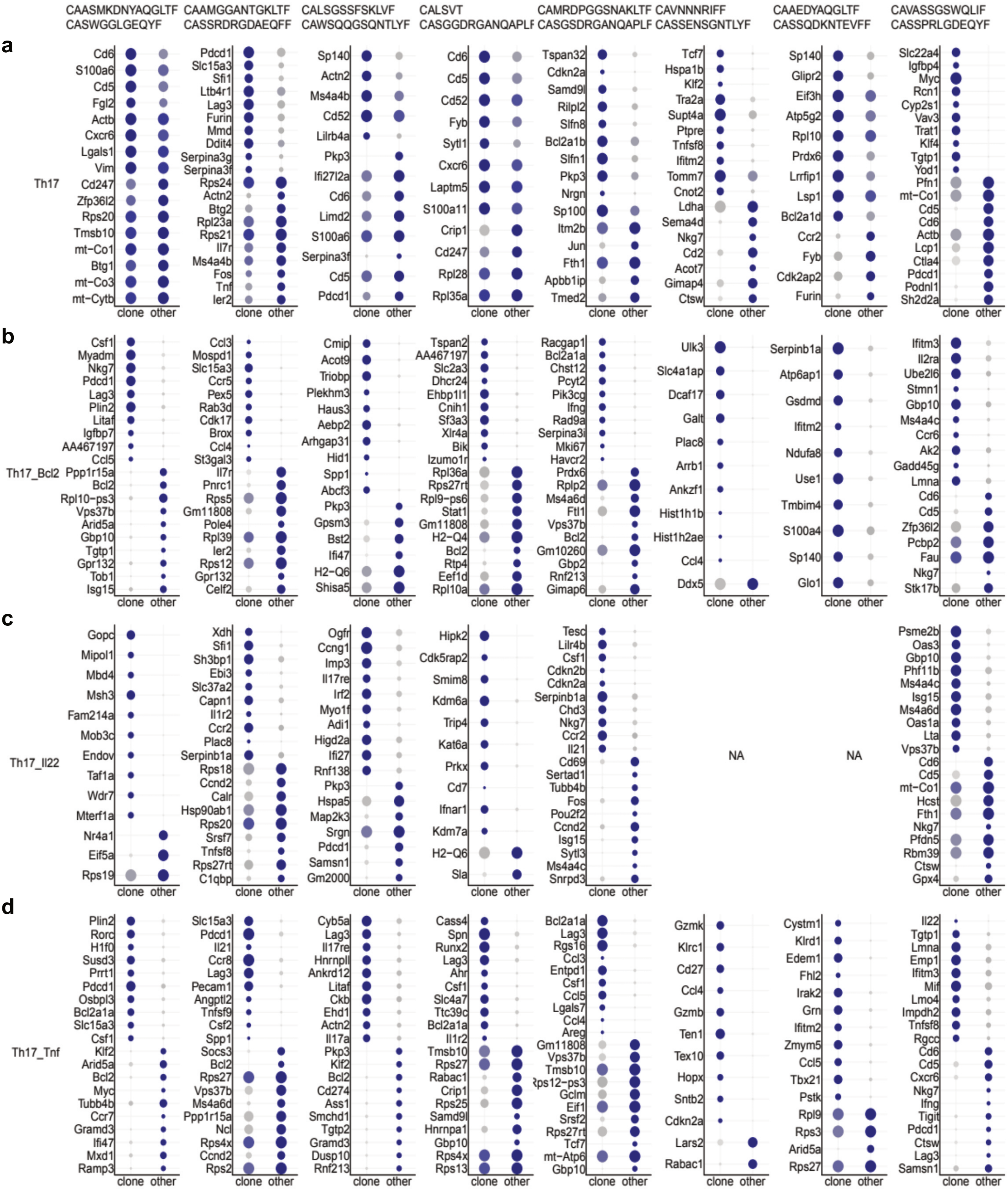
CAASMKDNYAQGLTF CASWGGLGEQYF
CAAMGGANTGKLTF CASSRDRGDAEQFF
CALSGGSSFSKLVF CAWSQQGSQNTLYF
CALSVT CASGGDRGANQAPLF
CAMRDPGGSNAKLTF CASGSDRGANQAPLF
CAVNNNRIFF CASSENSGNTLYF
CAAEDYAQGLTF CASSQDKNTEVFF
CAVASSGSWQLIF CASSPRLGDEQYF
a
Th17
Cd6 S100a6 Cd5 Fgl2 Actb Cxcr6 Lgals1 Vim Cd247 Zfp36l2 Rps20 Tmsb10 mt-Co1 Btg1 mt-Co3 mt-Cytb
Pdcd1 Slc15a3 Sfi1 Ltb4r1 Lag3 Furin Mmd Ddit4 Serpina3g Serpina3f Rps24 Actn2 Btg2 Rpl23a Rps21 Il7r Ms4a4b Fos Tnf Ier2
Sp140 Actn2 Ms4a4b Cd52 Lilrb4a Pkp3 Ifi27l2a Cd6 Limd2 S100a6 Serpina3f Cd5 Pdcd1
Cd6 Cd5 Cd52 Fyb Sytl1 Cxcr6 Laptm5 S100a11 Crip1 Cd247 Rpl28 Rpl35a
Tspan32 Cdkn2a Samd9l Rilpl2 Slfn8 Bcl2a1b Slfn1 Pkp3 Nrgn Sp100 Itm2b Jun Fth1 Apbb1ip Tmed2
Tcf7 Hspa1b Klf2 Tra2a Supt4a Ptprc Tnfsf8 Ifitm2 Tomm7 Cnot2 Ldha Sema4d Nkg7 Cd2 Acot7 Gimap4 Ctsw
Sp140 Glipr2 Eif3h Atp5g2 Rpl10 Prdx6 Lrrfip1 Lsp1 Bcl2a1d Ccr2 Fyb Cdk2ap2 Furin
Slc22a4 Igfbp4 Myc Rcn1 Cyp2s1 Vav3 Trat1 Klf4 Tgtp1 Yod1 Pfn1 mt-Co1 Cd5 Cd6 Actb Lcp1 Ctla4 Pdcd1 Podnl1 Sh2d2a
clone other
b
Th17_Bcl2
Csf1 Myadm Nkg7 Pdcd1 Lag3 Plin2 Litaf Igfbp7 AA467197 Ccl5 Ppp1r15a Bcl2 Rpl10-ps3 Vps37b Arid5a Gbp10 Tgtp1 Gpr132 Tob1 Isg15
Ccl3 Mospd1 Slc15a3 Ccr5 Pex5 Rab3d Cdk17 Brox Ccl4 St3gal3 Il7r Pnrc1 Rps5 Gm11808 Pole4 Rpl39 Ier2 Rps12 Gpr132 Celf2
Cmip Acot9 Triobp Plekhm3 Haus3 Aebp2 Arhgap31 Hid1 Spp1 Abcf3 Pkp3 Gpsm3 Bst2 Ifi47 H2-Q6 Shisa5
Tspan2 AA467197 Slc2a3 Dhcr24 Ehbp1l1 Cnih1 Sf3a3 Xlr4a Bik Izumo1r Rpl36a Rps27rt Rpl9-ps6 Stat1 Gm11808 H2-Q4 Bcl2 Rtp4 Eef1d Rpl10a
Racgap1 Bcl2a1a Chst12 Pcyt2 Pik3cg Ifng Rad9a Serpina3i Mki67 Havcr2 Prdx6 Rplp2 Ms4a6d Ftl1 Vps37b Bcl2 Gm10260 Gbp2 Rnf213 Gimap6
Ulk3 Slc4a1ap Dcaf17 Galt Plac8 Arrb1 Ankzf1 Hist1h1b Hist1h2ae Ccl4 Ddx5
Serpinb1a Atp6ap1 Gsdmd Ifitm2 Ndufa8 Use1 Tmbim4 S100a4 Sp140 Glo1
Ifitm3 Il2ra Ube2l6 Stmn1 Gbp10 Ms4a4c Ccr6 Ak2 Gadd45g Lmna Cd6 Cd5 Zfp36l2 Pcbp2 Fau Nkg7 Stk17b
clone other
c
Th17_Il22
Gopc Mipol1 Mbd4 Msh3 Fam214a Mob3c Endov Taf1a Wdr7 Mterf1a Nr4a1 Eif5a Rps19
Xdh Sfi1 Sh3bp1 Ebi3 Slc37a2 Capn1 Il1r2 Ccr2 Plac8 Serpinb1a Rps18 Ccnd2 Calr Hsp90ab1 Rps20 Srsf7 Tnfsf8 Rps27rt C1qbp
Ogfr Ccng1 Imp3 Il17re Irf2 Myo1f Adi1 Higd2a Ifi27 Rnf138 Pkp3 Hspa5 Map2k3 Srgn Pdcd1 Samsn1 Gm2000
Hipk2 Cdk5rap2 Smim8 Kdm6a Trip4 Kat6a Prkx Cd7 Ifnar1 Kdm7a H2-Q6 Sla
Tesc Lilr4b Csf1 Cdkn2b Cdkn2a Serpinb1a Chd3 Nkg7 Ccr2 Il21 Cd69 Sertad1 Tubb4b Fos Pou2f2 Ccnd2 Isg15 Sytl3 Ms4a4c Snrpd3
NA
NA
Psme2b Oas3 Gbp10 Phf11b Ms4a4c Isg15 Ms4a6d Oas1a Lta Vps37b Cd6 Cd5 mt-Co1 Hcst Fth1 Nkg7 Pfdn5 Rbm39 Ctsw Gpx4
clone other
d
Th17_Tnf
Plin2 Rorc H1f0 Susd3 Prrt1 Pdcd1 Osbpl3 Bcl2a1a Slc15a3 Csf1 Klf2 Arid5a Bcl2 Myc Tubb4b Ccr7 Gramd3 Ifi47 Mxd1 Ramp3
Slc15a3 Pdcd1 Il21 Ccr8 Lag3 Pecam1 Angptl2 Tnfsf9 Csf2 Spp1 Socs3 Bcl2 Rps27 Vps37b Ms4a6d Ppp1r15a Ncl Rps4x Ccnd2 Rps2
Cyb5a Lag3 Il17re Hnrnpll Ankrd12 Litaf Ckb Ehd1 Actn2 Il17a Pkp3 Klf2 Bcl2 Cd274 Ass1 Smchd1 Tgtp2 Gramd3 Dusp10 Rnf213
Cass4 Spn Runx2 Lag3 Ahr Csf1 Slc4a7 Ttc39c Bcl2a1a Il1r2 Tmsb10 Rps27 Rabac1 Crip1 Rps25 Samd9l Hnrnpa1 Gbp10 Rps4x Rps13
Bcl2a1a Lag3 Rgs16 Ccl3 Entpd1 Csf1 Ccl5 Lgals7 Ccl4 Areg Gm11808 Vps37b Tmsb10 ps12-ps3 Gclm Eif1 Srsf2 Rps27rt Tcf7 mt-Atp6 Gbp10
Gzmk Klrc1 Cd27 Ccl4 Gzmb Ten1 Tex10 Hopx Sntb2 Cdkn2a Lars2 Rabac1
Cystm1 Klrd1 Edem1 Fhl2 Irak2 Grn Ifitm2 Zmym5 Ccl5 Tbx21 Pstk Rpl9 Rps3 Arid5a Rps27
Il22 Tgtp1 Lmna Emp1 Ifitm3 Mif Lmo4 Impdh2 Tnfsf8 Rgcc Cd6 Cd5 Cxcr6 Nkg7 Ifng Tigit Pdcd1 Ctsw Lag3 Samsn1
clone other

Supplementary Figure Legends

Figure S1

Intranasal immunization with CTA1-3M2e-DD stimulates M2e-specific CD4 T cell resident memory cells in the lung

(a) In vitro cultures of splenocytes from i.n immunized mice were stimulated with M2e-recall peptide after depletion of CD4 or CD8 T cells. The impact of T cell depletion on M2e T cell proliferation was determined after 72h with 3H-thymidine incorporation for the last 6h and values are given for triplicate cultures in mean cpm ± SD of 3 mice and one representative experiment of two giving similar results is shown. (b) Mice (n=10) immunized i.n as in Fig. 1c developed local IgA antibodies specific for M2e which were detectable in their bronchioalveolar lavage at sacrifice and given in log_{10} titers± SD for indicated groups. (c) The virus titers in lungs of immunized mice (n=6) at 4 days post challange after CD4 or CD8 T cell depletion in vivo, as indicated and values are given as pfu/lung ± SD for each group. (d) Corresponding serum anti-M2e IgG2a log_{10} titers± SD to the total IgG titers given for i.n immunized mice prior to challenge infection. (e) Evidence that AP7 conjugated anti-CD45.1 mAb given i.v 5 min prior to sacrifice does not bind to M2e-tetramer specific cells by FACS. Furthermore, anti-CD44 and CD4 identifies resident memory CD4 T cells in the lungs while no cells are detectable in the blood (right panel).

Figure S2.

Single cell RNA-seq quality metrics and UMAP clustering.(a) Expression of T cell marker genes used to sort cells Cd3e, Cd3g, Thy1 and Cd4. Cd8a and Cd8b gene expression was not found in the dataset. (b) SNN graph overlay onto the UMAP embedding. Each line represents a connection between 2 cells. Cells are coloured by the clusters identified with Louvain. For practical reasons, only 30% of the lines are visualized. A graph abstraction with black lines for

each cluster is overlaid on top of the UMAP. Lines represent that >2% of the cluster cells are connected and the thickness indicates the percentage of connections between 2 clusters. (c) Quality metrics visualized as bar plots for day 3 and 8 of infection (left) or separated per cell cluster (right). Black lines represent the average values for each group.

Figure S3.

Functional classification of differentially expressed genes across all clusters. (a) Cytokines (b) Receptors (c) Trascription Factors and (d) other genes. The top 6 genes per clusters are shown, sorted by p-value and log differential detection percentage.

Figure S4

Expression of cluster-defining key marker genes projected onto the UMAP embedding of the M2e-binding CD4 T cells. (a) Naïve T cells, (b) T follicular helper cells (Tfh), (c) Foxp3-pos regulatory T cells (Treg), (d) Foxp3-neg regulatory T cells (Tr1), (e) T helper 1 cells (Th1), (f) Cytotoxic CD4 T cells (CTL, (g) Cells hosting interferon-stimulated genes (Isg), (h) Integrinbeta-1 transitional cells, (i) Archetypical T helper 17 cells, (j) Classical T helper 17 cells, (k) IL22^{+} T helper 17 cells (Th17:IL-22 or Th22). (l) Tnf^{+} T helper 17 cells (Th17:Tnf /“pathogenic”"Th17 cells) (m) Bcl2^{+} T helper 17 (Th17:Bcl2) (n) Cells expressing regulatory genes.

Figure S5.

Conserved up- (a) or down-regulated (b) genes in day 3 and day 8 M2e-specific Trm cells. Asterisks show whether that gene is differentilaly expressed in day 3 relative to day 8 samples (a) or day 8 relative to day 3 samples (b). Bar plots on the right represent how many clusters that the gene was found differentially expressed in.

Figure S6.

Differential VDJ gene usage for TCR-α and TCR-β CDR3-regions in M2e-tetramer binding cells (a) Heatmap of TCR-α pairings with complete VDJ gene sequences, (b) TCR-α pairings with V-chain sequences only or (c) TCR-α pairings with J-chain sequences only. (d) Heatmap of TCR-β pairings with complete VDJ gene sequences, (e) TCR-β pairings with V-chain sequences only or (f) TCR-β pairings with J-chain sequences only.

Figure S7.

TCR clonotype assessments using VDJ gene usage analysis for unique α and β chain CDR3-regions of M2e-tetramer binding cells. a) Distribution of the most expanded TCR clonotypes and b) their distribution in day 3 and day 8 samples with individual cells from each clonotype in the different functional subsets.

Figure S8

Differential gene expression of the eight most abundant M2e-specific TCR clonotypes in the four different Th17-subclusters . The unique clonotypes are listed above each column and the Th17 subclusters are listed in rows a-d.

Description of supplementary data files

Supplementary Data 1. Table

Supplementary Data 2. Table

Supplementary Data 3. Table

Supplementary Data 4. Table
